# Supplementary material for: Genomes from a medieval mass burial show Ashkenazi-associated hereditary diseases pre-date the 12th century
Source: Curr Biol. 2022 Oct 24;32(20):4350–4359.e6. doi: 10.1016/j.cub.2022.08.036 (PMC10499757; doi:10.1016/j.cub.2022.08.036)
Supplement: Document S1. Figures S1–S4 and Tables S1–S5 [file mmc1.pdf]

Current Biology, Volume *32*

Supplemental Information

Genomes from a medieval mass burial show Ashkenazi-associated hereditary diseases pre-date the 12th century

Selina Brace, Yoan Diekmann, Thomas Booth, Ruairidh Macleod, Adrian Timpson, Will Stephen, Giles Emery, Sophie Cabot, Mark G. Thomas, and Ian Barnes

A

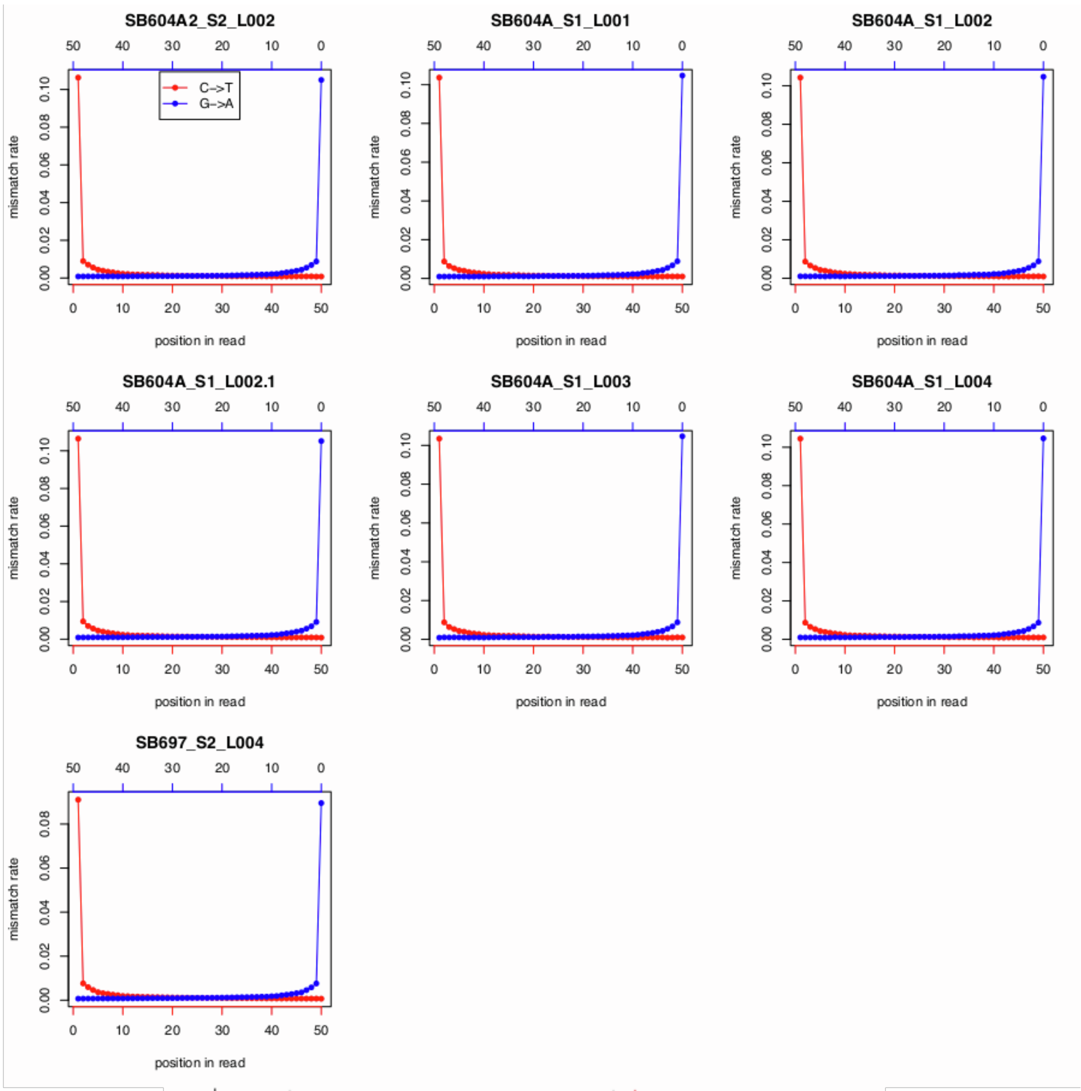
SB604A2_S2_L002
SB604A_S1_L001
SB604A_S1_L002
SB604A_S1_L002.1
SB604A_S1_L003
SB604A_S1_L004
SB697_S2_L004
C->T
G->A
mismatch rate
position in read

B

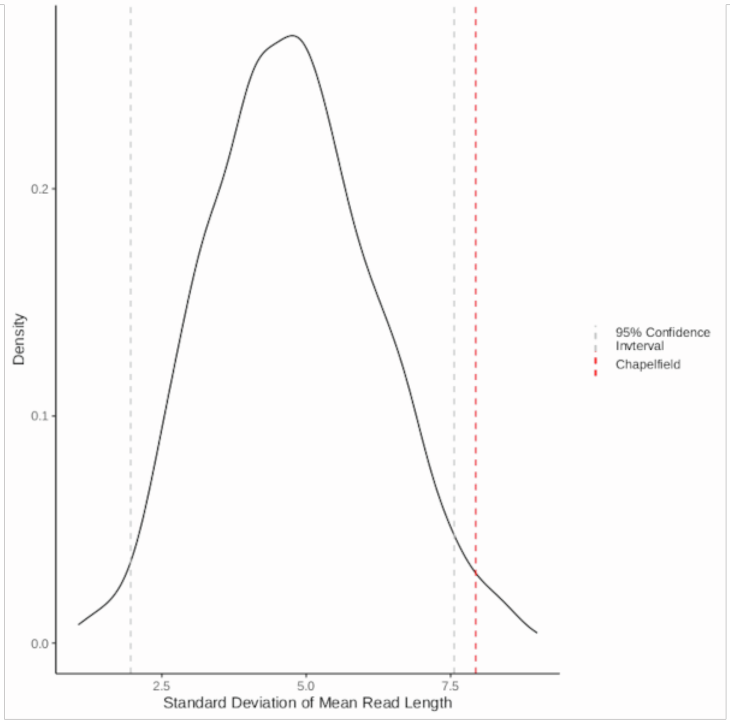
Density
Standard Deviation of Mean Read Length
95% Confidence Invterval
Chapelfield

Figure S1. Analysis of post-mortem damage patterns to confirm authenticity of aDNA, related to STAR Methods. (A) Misincorporation rates at DNA strand ends for seven sequencing libraries for the individual SB604, showing characteristic ‘ancient’ damage profiles in all libraries. These show cytosine to thymine and guanine to adenine misincorporation patterns at the first and last 50 base pairs of reads respectively for a subset of the sequenced libraries, confirming aDNA authenticity. Misincorporation rates were generated in ATLAS[S1]. (B) Standard deviation of mean read length for six randomly sampled ancient genomes compared with the Chapelfield individuals. This was investigated using the lambda parameter to estimate true fragment length[S2]; this indicates that depositional history is not a predictor of fragment length.

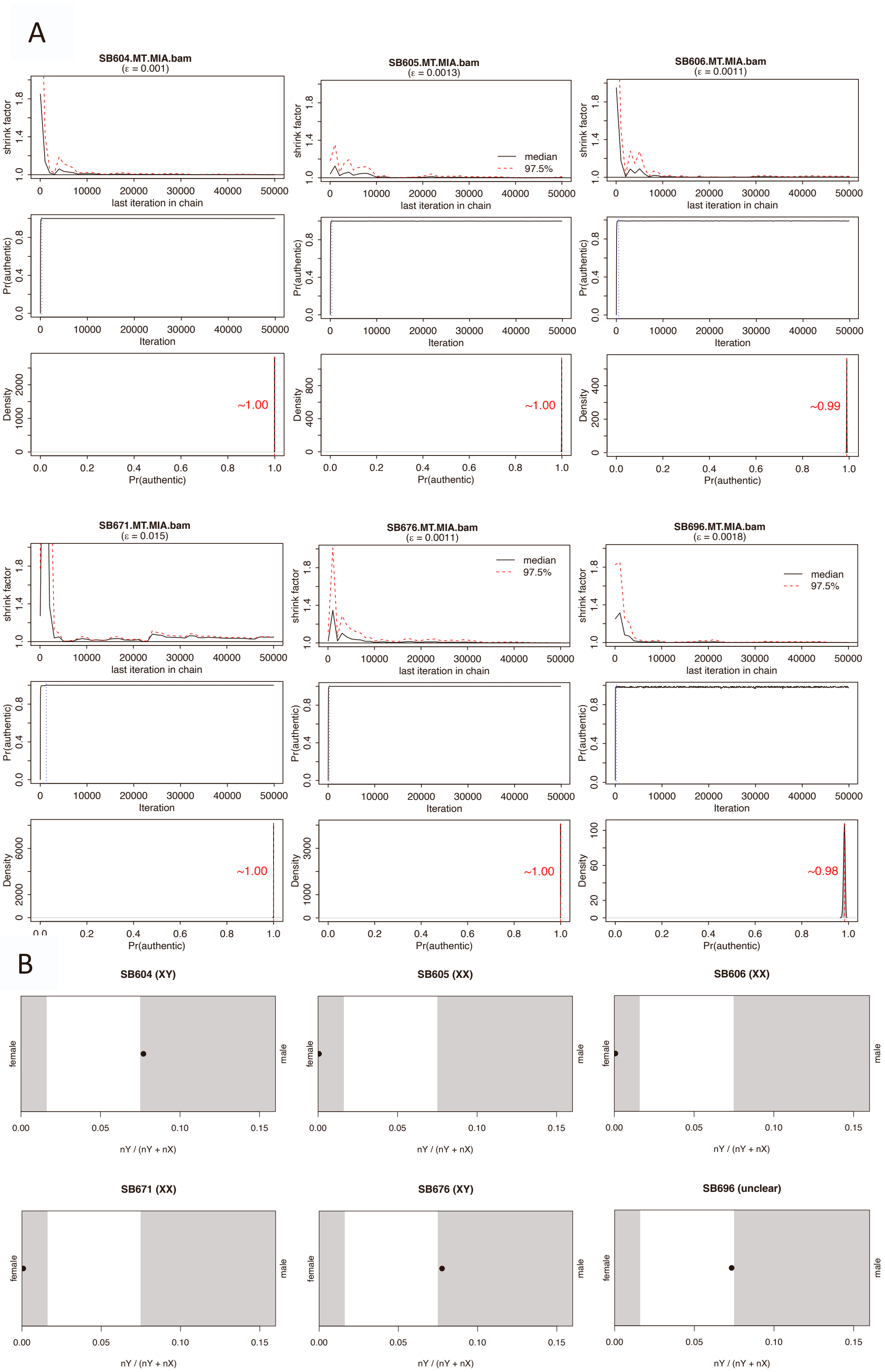
A
SB604.MT.MIA.bam
(ε = 0.001)
SB605.MT.MIA.bam
(ε = 0.0013)
SB606.MT.MIA.bam
(ε = 0.0011)
shrink factor
last iteration in chain
median
97.5%
Pr(authentic)
Iteration
Density
~1.00
~1.00
~0.99
SB671.MT.MIA.bam
(ε = 0.015)
SB676.MT.MIA.bam
(ε = 0.0011)
SB696.MT.MIA.bam
(ε = 0.0018)
~1.00
~1.00
~0.98
B
SB604 (XY)
SB605 (XX)
SB606 (XX)
SB671 (XX)
SB676 (XY)
SB696 (unclear)
female
male
nY / (nY + nX)

Figure S2. Basic bioinformatics analyses for mitochondrial sequence contamination and chromosomal sex, related to STAR Methods. (A) Results from mitochondrial contamination estimates for each sequenced individual. These indicate no mitochondrial sequence contamination, and were estimated through ContamMix[S3]. (B) Plots showing chromosomal sex of each individual based on ratios of X and Y chromosome sequences. These were predicted following the approach of Skoglund et al.[S4]. Shaded areas indicate female and male assignation; we also computed the R_x statistic from Mittnik et al.[S5], which confidently identified SB696 as male.

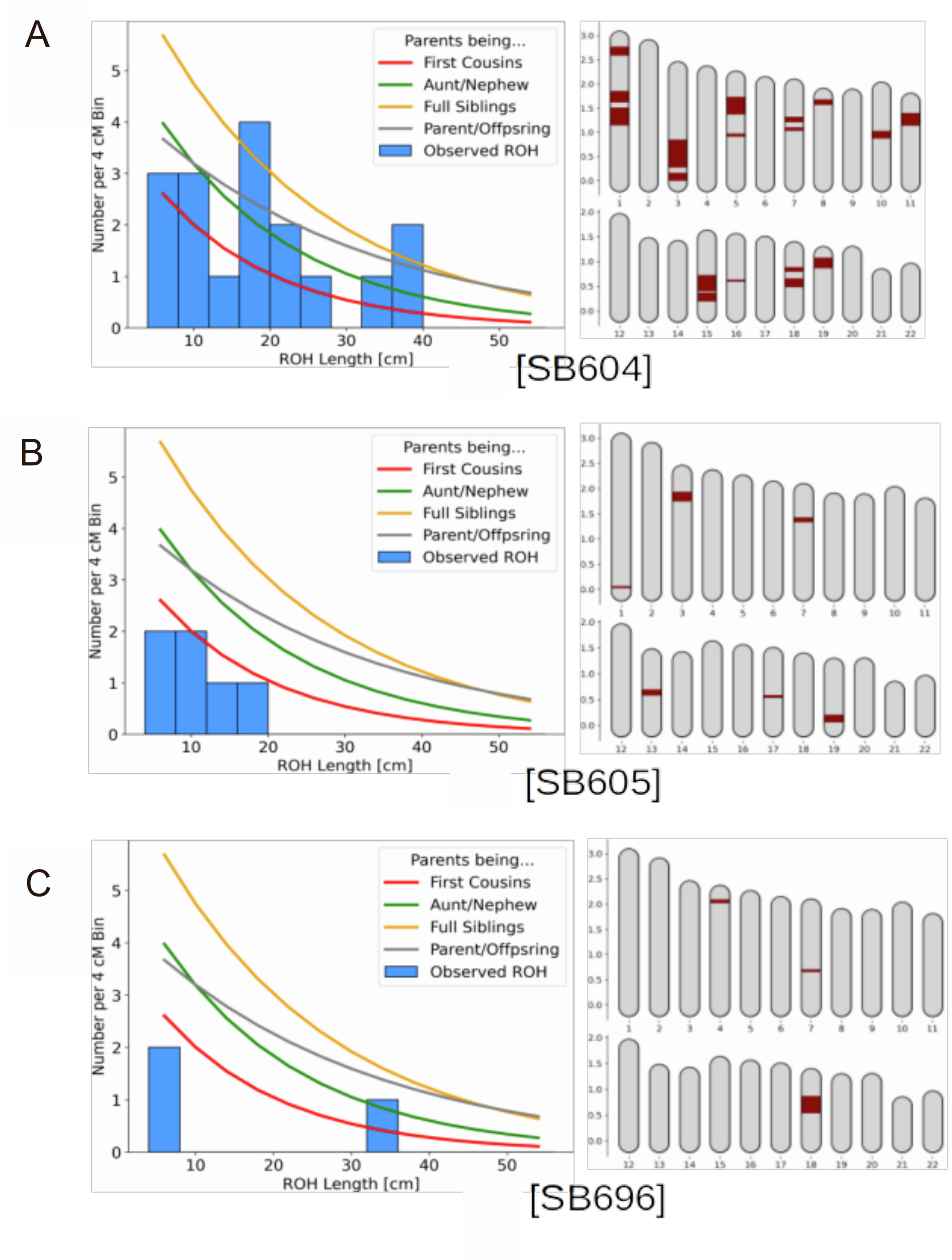

Figure S3. Runs of homozygosity inferred using hapROH, related to Figure 2. (A) Distribution of ROH lengths for SB604 compared with certain inbreeding scenarios (left), generated in hapROH[S6], and locations of large inferred ROHs on autosomes. The reference inbreeding scenarios overlain are generated automatically in hapROH based on calculations detailed in Ringbauer et al.[S6]. (B) As above for SB605. (C) As above for SB696.

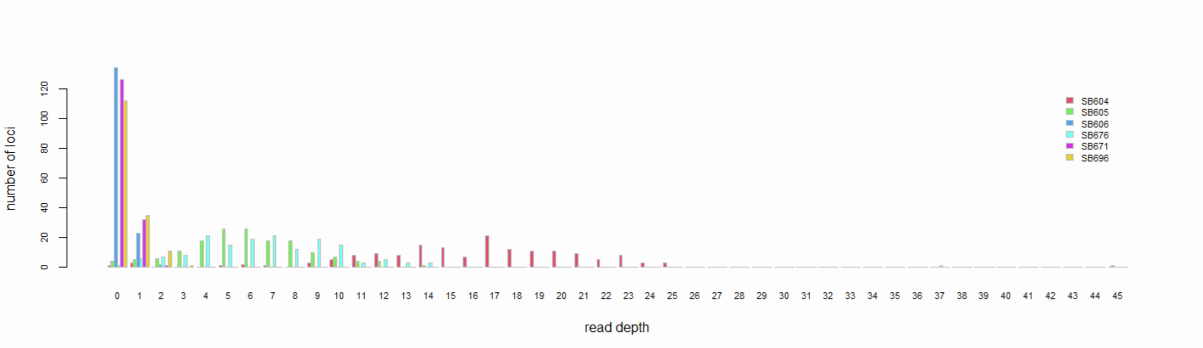

Figure S4. Read depth per disorder-associated loci for each sample, related to Figure 4 and STAR Methods. SB604 yielded the best read depth, averaging 16 reads per locus and zero reads at only 1 locus. SB606 yielded the poorest read depth, averaging 0.17 reads per locus with zero reads at 134 loci.

Sk Number (Deposit)	Lab Number	δ13C (AMS)	δ15N (AMS)	%C	%N	C:N Ratio	Radiocarbon Determination	Error
Sk 78	Wk16920	-18.3	13.14	45.5	16.2	3.28	875	34
Sk 62	Wk16919	-18.4	12.05	46.1	16.6	3.2	928	32
	SUERC-33391	-	-	-	-	-	910	30
	SUERC-33282	-	-	-	-	-	845	30
	SUERC-33281	-	-	-	-	-	850	30

Table S1. Radiocarbon date data, related to Figure 1C. This comprises Accelerator Mass Spectrometry (AMS) results for the total of five radiocarbon samples. Of these, two were reported by Emery et al.[S7] (Wk16920 and Wk16919) and the remaining three were commissioned by SHINE TV as part of the History Cold Case TV Series at the SUERC radiocarbon dating laboratory.

	Mitochondrial haplogroup	Y chromosome haplogroup
**SB604**	J1c5c1	J1a2a1a2d2b2a2b
**SB605**	H5c2	*NA*
**SB606**	H5c2	*NA*
**SB671**	H5c2	*NA*
**SB676**	H3w	E1b1b1b2a1b1a
**SB696**	U6a1b1b	T1a1a

Table S2. Uniparental haplogroups from HaploGrep2 and Yleaf, related to STAR Methods. Mitochondrial haplogroups were inferred using HaploGrep 2[S8] and Y chromosome haplogroups using Yleaf[S9].

	Mitochondrial haplogroup	Range	Quality	Global private mutations	Local private mutations	Assumed back mutations or missing
**SB604**	J1c5c1	1-16569	94.84%	3107T, 4490A, 4510G, 4687A, 5180, 8898A		4216, 7028, 8860
**SB605**	H5c2	1-16569	77.44%	3107T, 5177T, 7494A, 9081	3360, 5892	4769, 6776, 8860
**SB606**	H5c2	1-16569	78.69%	3107T, 4130A, 4134T, 4139A, 4141A, 4153T, 4165A, 7501G, 7541, 7634, 8919	4721, 8911	4769, 8860, 16304
**SB671**	H5c2	1-16569	72.34%	3107c, 4700, 5170, 5185, 5238, 5720, 5736, 5895, 5897, 6042A, 6107, 6877, 7096, 7504. 7550, 7552, 7566T, 7576, 7592, 7596A, 7627, 7732, 7756, 8897, 8903	1185, 4129, 4707, 4709, 7082, 7607	8860, 16304
**SB676**	H3w	1-16569	73.26%	3107T, 5177T, 7494A, 9081	3360, 5892	4769, 5892
**SB696**	U6a1b1b	1-16569	90.21%	4729A, 8001T	7696	4769, 7028, 8860, 11176

Table S3. Details of observed mitochondrial haplogroups and mutations, related to STAR Methods. These constitute results of classification using HaploGrep 2[S8] .

	PBlueEye	PIntermediateEye	PBrownEye	PRedHair	PBlondHair	PBrownHair	PBlackHair	PLightHair	PDarkHair	PVeryPaleSkin	PPaleSkin	PIntermediateSkin	PDarkSkin	PDarktoBlackSkin
**SB 604**	0.9110910019	0.05659703369	0.03231196441	0.9999541868	4.33E-05	1.33E-06	1.16E-06	0.9436118803	0.05638811966	0.02943904649	0.4400186053	0.5277118875	0.002826723683	3.74E-06
**SB 605**	0.000271927178	0.01339029227	0.9863377806	0.001123064907	0.06507180104	0.7585584683	0.1752466657	0.2407708189	0.7592291811	0.01111802015	0.05286550598	0.6327007046	0.2988146386	0.004501130685
**SB 676**	0.05032274533	0.1135511369	0.8361261178	0.004027658594	0.357854928	0.5652752736	0.07284213988	0.802023479	0.197976521	0.009700711401	0.3535418605	0.63433943	0.002399310288	1.87E-05

Table S4. Probabilities of phenotypes inferred with HirisPlexS, related to STAR Methods. Results are only reported for the three individuals with sufficient genomic analysis for this approach.

**Sample**	***f***	**Avg. autosomal depth**
SB604	0.2064325334	13.81x
SB605	0.09459731039	4.78x
SB606	0	0.16x
SB671	0	0.18x
SB676	0.1245771792	6.03x
SB696	0	0.31x

Table S5. Inbreeding coefficients (*f*) calculated using estimates of homozygous-by-descent segments in PLINK, related to Figure 2 and STAR Methods. For this analysis, only SB604 has sufficient genomic coverage for confident inferences to be drawn.

Supplemental References

S1. Link, V., Kousathanas, A., Veeramah, K., Sell, C., Scheu, A., and Wegmann, D. (2017). ATLAS: Analysis Tools for Low-depth and Ancient Samples. bioRxiv, 105346.

S2. Kistler, L., Ware, R., Smith, O., Collins, M., and Allaby, R.G. (2017). A new model for ancient DNA decay based on paleogenomic meta-analysis. Nucleic Acids Res. *45*, 6310–6320.

S3. Fu, Q., Mittnik, A., Johnson, P.L.F., Bos, K., Lari, M., Bollongino, R., Sun, C., Giemsch, L., Schmitz, R., Burger, J., et al. (2013). A revised timescale for human evolution based on ancient mitochondrial genomes. Curr. Biol. *23*, 553–559.

S4. Skoglund, P., Storå, J., Götherström, A., and Jakobsson, M. (2013). Accurate sex identification of ancient human remains using DNA shotgun sequencing. J. Archaeol. Sci. *40*, 4477–4482.

S5. Mittnik, A., Wang, C.-C., Svoboda, J., and Krause, J. (2016). A Molecular Approach to the Sexing of the Triple Burial at the Upper Paleolithic Site of Dolní Věstonice. PLoS One *11*, e0163019.

S6. Ringbauer, H., Novembre, J., and Steinrücken, M. (2021). Parental relatedness through time revealed by runs of homozygosity in ancient DNA. Nat. Commun. *12*, 5425.

S7. Emery, G., Dobson, D., Hoggett, R., and Whitmore, D. (2010). A Medieval Mass Grave on the site of the Chapelfield Shopping Centre, Norwich (NAU Archaeology).

S8. Weissensteiner, H., Pacher, D., Kloss-Brandstätter, A., Forer, L., Specht, G., Bandelt, H.-J., Kronenberg, F., Salas, A., and Schönherr, S. (2016). HaploGrep 2: mitochondrial haplogroup classification in the era of high-throughput sequencing. Nucleic Acids Res. *44*, W58–63.

S9. Ralf, A., Montiel González, D., and Zhong, K. (2018). Yleaf: software for human Y-chromosomal haplogroup inference from next-generation sequencing data. Mol. Biol.
