# Supplementary material for: Genomes from a medieval mass burial show Ashkenazi-associated hereditary diseases pre-date the 12th century
Source: Curr Biol. 2022 Oct 24;32(20):4350–4359.e6. doi: 10.1016/j.cub.2022.08.036 (PMC10499757; doi:10.1016/j.cub.2022.08.036)
Supplement: Document S2. Article plus supplemental information [file mmc3.pdf]

Current Biology

Article

Genomes from a medieval mass burial show Ashkenazi-associated hereditary diseases pre-date the 12th century

Highlights

- Medieval English human remains are source for earliest Jewish genomes
- Population continuity between 12th century and modern Ashkenazi Jews
- Four alleles associated with genetic disorders in Ashkenazi Jews observed
- Founder event elevating Jewish genetic disorder frequencies predates 12th century

Authors

Selina Brace, Yoan Diekmann, Thomas Booth, ..., Sophie Cabot, Mark G. Thomas, Ian Barnes

Correspondence

m.thomas@ucl.ac.uk (M.G.T.), i.barnes@nhm.ac.uk (I.B.)

In brief

Brace et al. find that human remains recovered from a medieval well in Norwich, UK, have strong genetic affinities with modern-day Ashkenazi Jews and were likely victims of recorded antisemitic violence. The presence of Ashkenazi-associated genetic disease alleles indicates a pre-12th century population founder event.

Check for updates

Brace et al., 2022, Current Biology *32*, 4350–4359
October 24, 2022 © 2022 The Authors. Published by Elsevier Inc.
https://doi.org/10.1016/j.cub.2022.08.036

CellPress

CellPress
OPEN ACCESS

Current Biology

Article

Genomes from a medieval mass burial show Ashkenazi-associated hereditary diseases pre-date the 12th century

Selina Brace,[1,11] Yoan Diekmann,[2,3,11] Thomas Booth,[1,4,8,9,11] Ruairidh Macleod,[1,2,5,11] Adrian Timpson,[2] Will Stephen,[1] Giles Emery,[6] Sophie Cabot,[7] Mark G. Thomas,[2,10,*] and Ian Barnes[1,12,*]
[1]Department of Earth Sciences, The Natural History Museum, Cromwell Road, London SW7 5BD, UK
[2]Research Department of Genetics, Evolution and Environment, University College London, Gower Street, London WC1E 6BT, UK
[3]Palaeogenetics Group, Institute of Organismic and Molecular Evolution (iomE), Johannes Gutenberg-University Mainz, 55099 Mainz, Germany
[4]Francis Crick Institute, London NW1 1AT, UK
[5]Department of Archaeology, University of Cambridge, Downing Street, Cambridge CB2 3DZ, UK
[6]Norvic Archaeology, 7 Foxburrow Road, Norwich NR7 8QU, UK
[7]Norfolk Record Office, The Archive Centre, Martineau Lane, Norwich, Norfolk NR1 2DQ, UK
[8]UCL Genetics Institute, University College London, London, UK
[9]Twitter: @Boothicus
[10]Twitter: @mt_genes
[11]These authors contributed equally
[12]Lead contact
*Correspondence: m.thomas@ucl.ac.uk (M.G.T.), i.barnes@nhm.ac.uk (I.B.)
https://doi.org/10.1016/j.cub.2022.08.036

SUMMARY

We report genome sequence data from six individuals excavated from the base of a medieval well at a site in Norwich, UK. A revised radiocarbon analysis of the assemblage is consistent with these individuals being part of a historically attested episode of antisemitic violence on 6 February 1190 CE. We find that four of these individuals were closely related and all six have strong genetic affinities with modern Ashkenazi Jews. We identify four alleles associated with genetic disease in Ashkenazi Jewish populations and infer variation in pigmentation traits, including the presence of red hair. Simulations indicate that Ashkenazi-associated genetic disease alleles were already at appreciable frequencies, centuries earlier than previously hypothesized. These findings provide new insights into a significant historical crime, into Ashkenazi population history, and into the origins of genetic diseases associated with modern Jewish populations.

INTRODUCTION

In 2004 construction workers excavating land in central Norwich, UK, as part of the Chapelfield shopping center development recovered human skeletal elements from their spoil.[1] Subsequent archaeological investigations led to the discovery and excavation of a probable well containing the commingled remains of at least seventeen people. The stratigraphic position of the remains, their completeness, and state of articulation suggested that they had all been deposited in a single event shortly after their death. The overrepresentation of subadults and the unusual location of the burial outside of consecrated ground suggested that they may have been victims of a mass fatality event such as famine, disease, or mass murder.

Pottery sherds from the well were dated typologically to 12th–14th centuries CE, and two initial radiocarbon determinations on the skeletal remains placed these in the 11th–12th centuries.[1] The most prominent historically attested mass death in Norwich within this date range was in 1190 CE when members of the Jewish community were killed during antisemitic riots precipitated by the beginning of the Third Crusade,[2] although the number of individuals killed is unclear.[3] Norwich had been the setting for a previous notable event in the history of medieval antisemitism when, in 1144 CE, the family of William of Norwich claimed that local Jews were responsible for his murder, an argument taken up by Thomas of Monmouth through the first documented invocation of the blood libel myth. This represents the beginnings of an antisemitic conspiracy theory that persists up to the present day.[4] The possibility that the remains found at the Chapelfield well site were those of the victims of antisemitic violence is given further support by the site's location just to the south of the medieval Jewish quarter of the city.[1,5] However, no additional archaeological evidence linked the human remains to a specific historical event or group of people. During the High Medieval period (ca. 1000–1300 CE), Norwich witnessed a number of outbreaks of large-scale violence,[6] and additional data were therefore required to test the hypothesis that these individuals were of Ashkenazi Jewish descent.

 © 2022 The Authors. Published by Elsevier Inc.
This is an open access article under the CC BY license (http://creativecommons.org/licenses/by/4.0/).

Judaism is a shared religious and cultural identity, with endogamous marriage practices and distinctive diasporic histories of communities worldwide, particularly a Levantine origin and complex history of migrations over the last ~2.5 millennia. Present-day Ashkenazim are descendants of medieval Jewish populations with histories primarily in northern and eastern Europe. As a result, they carry distinctive ancestries,[7–10] and Jewish and non-Jewish medieval individuals living in the same regions would likely show characteristic patterns of genetic variation.[11]

Hereditary disorders in Ashkenazi Jewish populations have been the focus of considerable medical research,[12–16] with genetic screening now commonplace to mitigate risks.[17] Their prevalence is generally attributed to strong genetic drift during Ashkenazi population bottlenecks,[10,18–23] coupled with high endogamy,[7,11,24] although other processes such as heterozygote advantage have been proposed.[25,26] Candidate population bottlenecks include the phase of dispersion following the destruction of the Second Temple in 70 CE, the formation of Ashkenazi communities in northern Europe during the medieval period, antisemitic persecution arising from the Crusades, unfounded reprisals for the Black Death, and the movement from western and central Europe to eastern Europe that preceded rapid population growth from the 15th to 18th centuries.[19,21,27,28]

No genomes from known Jewish individuals are currently available from the medieval period or earlier, largely because exhumation and scientific testing of Jewish remains are prohibited. Such data could inform on the migration and admixture histories of Jewish populations. Furthermore, the presence of any pathogenic variants would provide valuable clues to the origins and spread of Ashkenazim-associated genetic disorders. Here, we examine results from radiocarbon dating and genetic analyses of the Chapelfield individuals to better establish who they were, when they died, and the nature of their death and burial, and identify potential broader implications for Ashkenazim population history and genetics.

RESULTS

Radiocarbon dating

The two previously published radiocarbon dates[1] were supplemented by three further radiocarbon dates obtained by directly sampling the human remains (Method details: Radiocarbon dating). All five radiocarbon dates were consistent with each other (Acomb = 75.8), so they were calibrated and modeled as a single event using the OxCal function *Combine*.[29,30] This indicated that the bodies were deposited 1161–1216 calCE (calibrated radiocarbon years in the CE; 95% confidence) or 1165–1207 calCE (68% confidence). This date range is consistent with the only historically attested antisemitic massacre in Norwich in 1190 CE (Figure 1). However, this range also encompasses the so-called Great Revolt of 1174 CE when many people were killed during the sack of Norwich by Hugh Bigod.[6]

Ancient DNA

NGS libraries from 25 skeletal elements were screened, and nine libraries from six individuals were selected for higher coverage sequencing on the basis of endogenous DNA content > 4.5% (Data S1A). This resulted in autosomal average coverage (average read depth) per individual ranging from 0.16× to 13.81× and a mean read length of 68 bp from the six individuals (Data S1B). In addition to established methods for authenticating aDNA,[31,32] we studied DNA molecule degradation using the lambda parameter to estimate true fragment length[33] and compared results with a depositionally varied panel of ancient genomes (Method details: DNA fragmentation). We found significant variation in DNA fragmentation among the sequenced Chapelfield samples, indicating this cannot be predicted by depositional history.

Familial relationships

We inferred familial relationships among the Chapelfield group on the basis of pairwise relatedness coefficients and summary statistics (Method details: Inferring familial relationships and inbreeding). From this, it was inferred that three individuals were full-sibling sisters: SB606 (from Deposit Sk 75), a 10- to 15-year-old; SB671 (Deposit Sk 78), a young adult; and SB605 (Deposit Sk 69), a 5- to 10-year-old (Data S1G). These sisters were found to share the mitochondrial haplotype H5c2. In addition, SB696 appears more distantly related to this group, and SB676 is in turn distantly related to SB696.

Individual SB604 had multiple long runs of homozygosity (RoHs) comprising large portions of many chromosomes (Figures 2 and S3), and an inbreeding coefficient (0.21) close to that expected for the offspring of a first-order union. The very long RoHs (up to approximately 40 cM) identified in this individual indicate a very recent inbreeding event. Additionally, the proportions of long RoHs in SB676 and SB605 are consistent with their parents having been second-degree relatives (Figure S3). We exclude the possibility that the observed RoHs are entirely explained by low effective population size, as we would expect the distribution of RoH lengths to show an excess of short RoHs, which we do not observe (Figure 2).

Genetic ancestry

Present-day Ashkenazim are primarily descended from diasporas who settled in various parts of northwestern and, later, central and eastern Europe through the medieval period.[34] As such, they represent the present-day population that we would expect to be genetically most similar to Jewish individuals in medieval England. In addition, historical sources indicate that the Norwich Jewish community were descendants of Ashkenazi Jews from Rouen, Normandy, who were invited to England by William the Conqueror after 1066 CE.[35] We therefore explored the genetic affinities of the six Chapelfield individuals using principal component analysis (Figure 3) and tested whether modern Ashkenazi ancestry is better explained by the ancestry of the Chapelfield assemblage or a mixture of modern populations acting as proxies for ancient admixture components (e.g., Middle Eastern and Southern and Eastern European). We inferred ancestry proportions for modern Ashkenazi with *qpAdm*,[36] with Chapelfield, Turkish Jews, Sicilian, French, and Polish as potential sources, and found the best model to be one of 100% Chapelfield (p = 0.65; Data S1I; by convention values below 0.01 indicate a poor fit). We also modeled Chapelfield ancestry as a mixture of modern populations, which we use as proxies for hypothesized ancestry components: Turkish Jews, Sicilian, French, and Polish. We estimate a mixture of ~33%, ~67%, ~0%, and ~0%, respectively (p = 0.88). These results are consistent with a previous

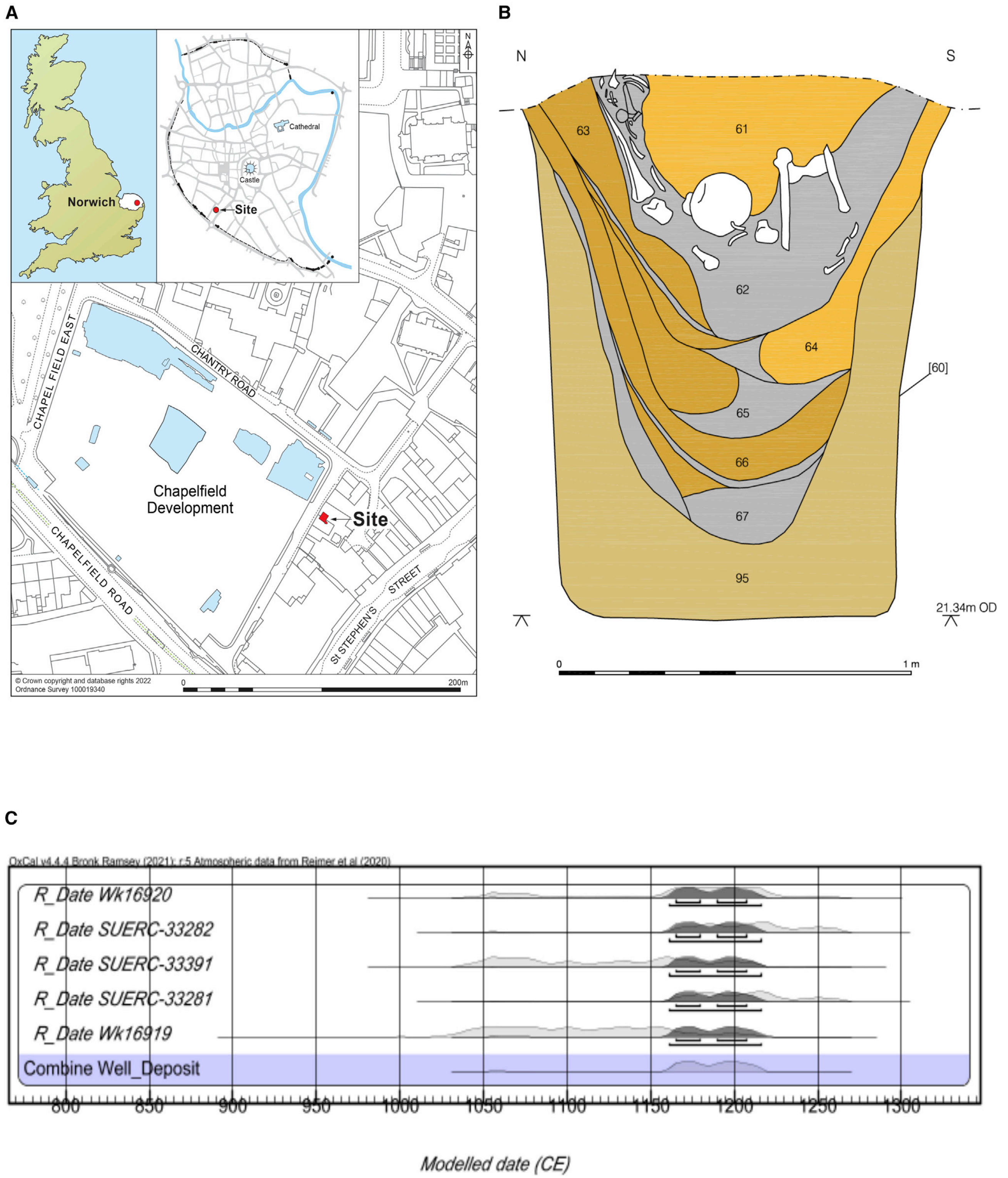

Figure 1. Details of the archaeological and radiocarbon information from the Chapelfield well deposit

(A) Location of the site in Norwich, UK.

(B) West-facing vertical section drawing of the Chapelfield well shaft.[1]

(C) Probability distribution for the date of deposition of the human remains based on 5 radiocarbon dates modeled as a single event using the *Combine* function in OxCal 4.4 and the IntCal20 curve. 1165–1179 CE (31.4% probability), 1190–1207 (36.8% probability), and 1161–1216 CE (95.4% probability). χ^2 test, df = 4, T = 4.882 (5% 9.488); agreement, n = 5; Acomb = 75.8% (An = 31.6%),[29,30] from isotopic data in Table S1.

See also Method details: Radiocarbon dating.

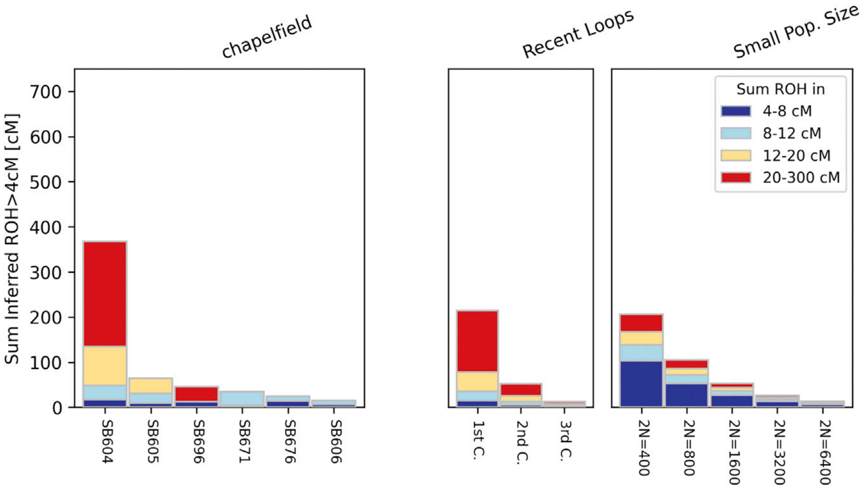

Figure 2. Runs of homozygosity inferred among the Chapelfield individuals using hapROH

The stacked plot on the right shows total lengths of RoHs for different length categories in the six sequenced Chapelfield individuals. Plots on the right show expected sum of RoH lengths for close kin ("Recent Loops") and under small population size scenarios. Detailed plots of RoH length distributions and autosome locations for SB604, SB605, and SB696 are provided in Figure S3, with comparisons indicated for certain recent inbreeding scenarios.

demographic model,[37] which places the introgression of Eastern European ancestry after the date of these individuals.

Uniparental haplogroup calls for all Chapelfield individuals also support genetic affinities to the Near East and in some cases to Ashkenazi Jewish populations (Method details: Sex and uniparental haplogroups). Specifically, Ashkenazi Jews form the majority of modern carriers for mitochondrial haplogroup H5c2,[39,40,41] in particular the back mutation at 16304, observed in the three sisters SB605, SB606, and SB671 (Table S3). The Y chromosome haplotype of SB676 (E1b1b1b2a1b1a) is within haplogroup E-M34, which is common in semitic language speakers and has a frequency of 11.7% among Ashkenazim for the parent haplogroup E-M12332. Similarly, the parent subclades of SB604 (J1a2a1a2d2b2) and SB696 (T1a1a) are particularly associated with Levantine ancestry.[42,43]

In order to temporally resolve genomic affinities, we estimated coalescence rates using *Colate*[44] based on allele ages inferred from a genealogy of a diverse set of modern populations.[45] In panmictic population models, intra-group coalescence rates are expected to be inversely related to effective population size. Between groups, they can be interpreted as a function of the intensity of gene flow prior to the coalescence event. They are therefore informative on population size history and demographic processes like admixture.

In the epoque roughly corresponding to 119 BCE to 1140 CE (Figure 4A), we find that Europeans are separated from modern Ashkenazi Jews and Middle Eastern individuals on the first axis of variation, with Chapelfield individuals in between but closer to Europeans. The second dimension sets modern Ashkenazi Jews apart, and to a lesser extent the Chapelfield individuals. Figure 4B summarizes the same pairwise coalescence rates by showing inter- and intra-group average pairwise coalescence rates (apCRs). Inter-group apCRs mirror the patterns of the first dimension of the multidimensional scaling (MDS) plot, while intra-group apCRs are highest in modern Ashkenazi Jews, followed by Chapelfield individuals, and can be seen as reflecting the variation shown in the second dimension of the MDS plot. The Chapelfield individuals have the highest inter-group apCR with modern Ashkenazi Jews. The relative ranking of apCRs for the older epoque roughly from 3278 BCE to 119 BCE shown in Figure 4C are similar to those in the later epoque, with one difference being approximately equal intra-group apCRs in Ashkenazi Jews and Chapelfield individuals.

We interpret these patterns as showing (1) some degree of population continuity between Chapelfield individuals and modern Ashkenazi Jews, consistent with the *qpAdm* results; (2) modern Ashkenazi Jews being a mixture of Middle Eastern and European populations, as, for example, found by Xue et al.[37] and consistent with the *qpAdm* results; (3) both modern Ashkenazi Jews and Chapelfield individuals having smaller effective population sizes than pan-Middle Eastern and -European populations, which is not sufficient to infer but consistent with a potential population bottleneck in Ashkenazim before 1140 CE; and (4) modern Ashkenazim having experienced additional bottlenecks or increased endogamy after 1140 CE.

Phenotypes and genetic disorders

Three individuals have sufficient genotyped SNPs to pass the threshold for HirisPlex[46] pigmentation phenotype inference (Method details: Inferring pigmentation phenotypes; Table S4). Two individuals were inferred to have had brown eyes, one with "dark" and one with "light" hair (SB605 and SB676, respectively), while the 0- to 3-year-old boy (SB604) was inferred to have had blue eyes and red hair, the latter of which is associated with historical stereotypes of European Jews.[47]

We examined the six Chapelfield genomes for variants associated with hereditary diseases in Ashkenazi Jews[10,16,48–50] at 159 loci. Previous studies have attributed the high frequencies of certain genetic disease alleles in Ashkenazi Jews principally to high rates of drift during population bottlenecks, variously hypothesized to have occurred around 1,100–1,400 CE,[21] ca. 900 CE,[19] ~1,300 CE,[24] 33 generations ago,[51] or 30 generations ago.[16] Because the Chapelfield individuals lived prior to, or at the start of these hypothesized bottlenecks, we would not expect the comparatively high frequencies of modern Ashkenazim-associated disease alleles to have been reached in the population to which they belonged. Rather, we expect the population at this time to have disease allele frequencies that are more typical of modern European populations.

Across the 159 Ashkenazi-associated genetic disorder loci considered, we amassed a total of 4,755 reads for the six Chapelfield individuals. Of these, 45 reads from 35 loci were genetic disorder alleles (one locus had 4 reads, two loci had 3 reads

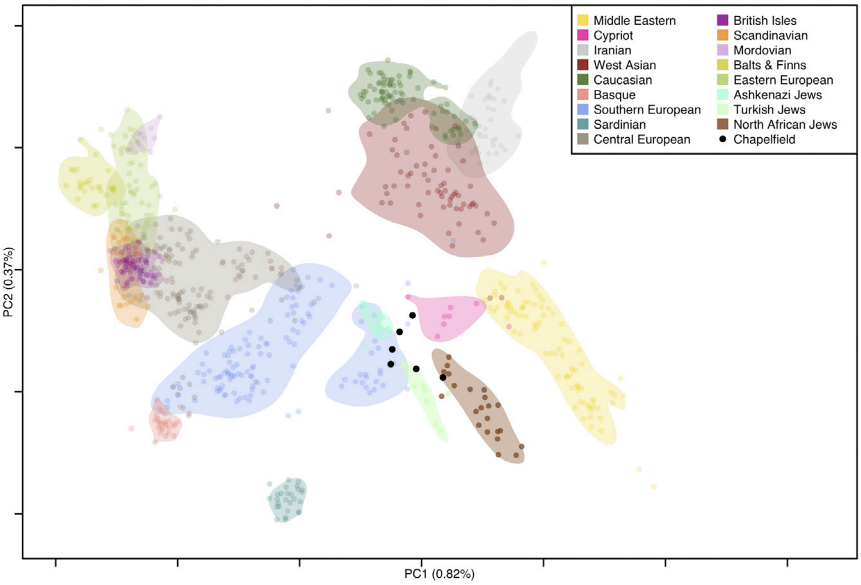

Figure 3. Principal component analysis (PCA) exploring the genetic affinities of the six Chapelfield individuals

We projected the six Chapelfield genomes on a PCA defined by variation among modern western Eurasian population samples, including modern Jewish individuals. All six Chapelfield individuals project well away from present-day British samples, as well as northern Europeans more generally. Instead, they partially overlap with Southern Europeans, close to Cypriots, modern Ashkenazi, Turkish, and North African Jews. These results are consistent with the Chapelfield individuals having Jewish ancestry (cf. Kopelman et al.[38]).

each, three loci had 2 reads each, and 29 loci had 1 read each). However, it is important to note that some of these reads will be type 1 errors, falsely indicating the presence of a disease allele. Therefore, we simulated datasets assuming both the modern European (non-Finnish) and modern Ashkenazi population allele frequencies in the gnomAD database,[52] to explore how many disease allele reads should be expected in our sample at different read error rates. We sampled A,C,G,T nucleotides (at each locus, for each individual) from a multinomial distribution, using the observed total read depth as the number of trials. To account for read errors, we introduce a read error parameter α (assumed constant across all loci) to adjust the gnomAD allele frequencies, which were

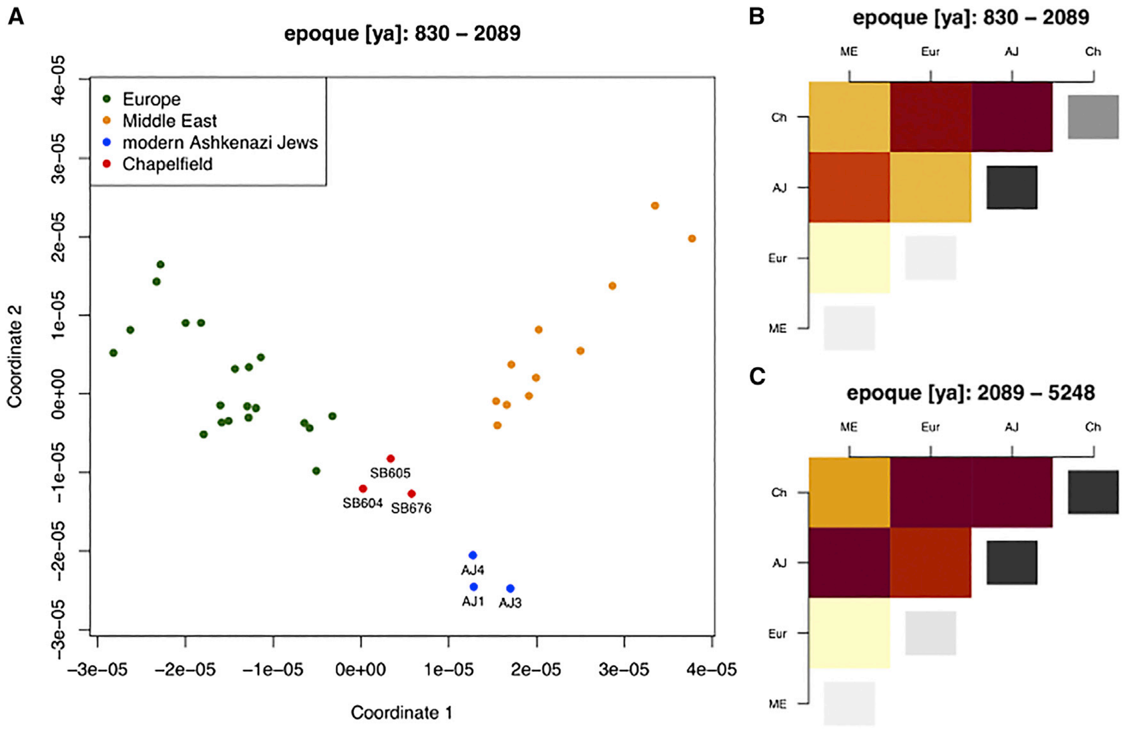

Figure 4. Pairwise coalescence rates between European, Middle Eastern, modern Ashkenazi, and Chapelfield individuals

(A) Multidimensional scaling (MDS) of pairwise coalescence rates. Rates between the chromosomes of a single individual are not included.

(B and C) Heatmaps of average pairwise coalescence rates (apCRs) between and within groups of individuals. Color code for inter-group comparisons goes from light yellow (low apCR) to dark red (high apCR); for intra-goup apCR from light gray (low apCR) to black (high apCR). years ago, ya; Middle Eastern, ME; European, Eur; modern Ashkenazi Jews, AJ; Chapelfield, Ch.

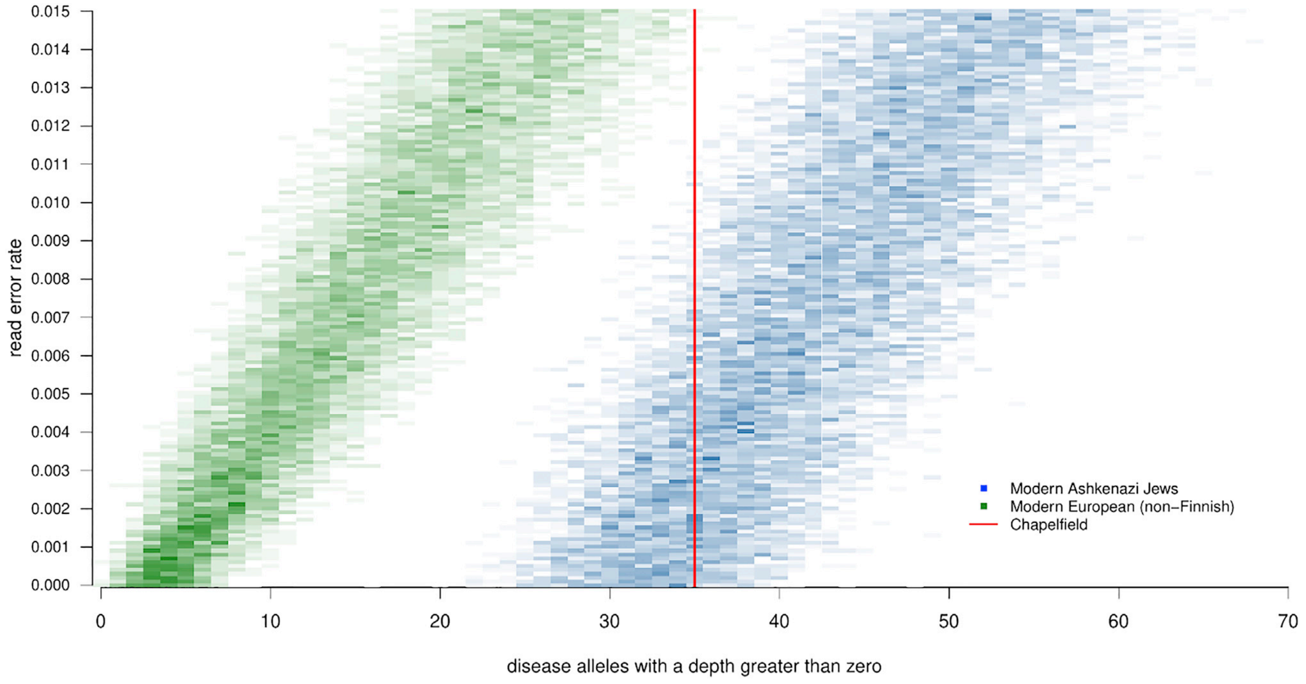

Figure 5. Heatmap of simulation results for genetic disorders

Comparison of test statistic (number of disease alleles with a read depth > 0) of observed Chapelfield data with 22,500 simulations under the assumption of modern Ashkenazi Jewish population allele frequencies, and modern European (non-Finnish) population allele frequencies, across a range of plausible read error rates. Methods underlying this analysis are described in Method details: Analysis of Ashkenazi-associated Mendelian disorders, and observed read depth per loci in the Chapelfield samples are shown in Figure S4.

then used as the multinomial probabilities. As a simple test statistic to compare our observed data with simulations, we use the total number of disease alleles with one or more reads. Figure 5 illustrates that across a range of plausible error rates (0% to 1.5%), our test statistic for the Chapelfield data is typically expected given modern Ashkenazi Jewish population allele frequencies (one-tail test for greater or equal to 35: p = 0.8143), but unlikely given modern European (non-Finnish) frequencies (one-tail test for greater or equal to 35, p = 0.0048).

To explore this further, we formulated a likelihood function to calculate the exact probability of the six individuals' observed allele reads at the 159 disease loci, given the allele frequencies of any proposed population. The likelihood function utilizes the read error parameter, summing the probability of the observed data for all 10^6 permutations of the ten possible genotypes at a single locus. We assume loci are independent; therefore, the overall probability is simply the product of each locus-specific probability. This allows us to calculate the likelihood of either the modern European (non-Finnish) or modern Ashkenazi populations, given the data. To further account for uncertainty in the allele frequencies of these modern populations, we use the gnomAD[52] counts of alleles as parameters in a Dirichlet distribution (with a uniform prior) to sample across possible population allele frequencies. Figure 6 illustrates these likelihoods under 5,000 random samples, each with a different read error rate sampled from a uniform distribution between 0% and 1.5%. The maximum likelihood read error rate estimates are notably similar (0.87% and 0.94%, respectively), and crucially these results show that the data are 4,615 times more probable under a model that these individuals were sampled from the modern Ashkenazi population than they were sampled from the modern non-Finnish European population. This approach assumes the six individuals are randomly sampled from either population. Further assessment of the effect of this assumption given that three individuals are siblings suggests that in the case of these data our assumption has a conservative effect on the likelihood ratio (Method details: Analysis of Ashkenazi-associated Mendelian disorders).

The likelihood ratios (LRs) at the vast majority of loci (155 of 159) have little influence on this (mean = 0.967, SD = 0.054, range 0.777 to 1.125), since loci with no reads are equally probable (certain) under either population model, and those with only non-pathogenic allele reads are marginally more probable under the European population in which the pathogenic alleles are at a lower frequency. Instead, the substantial overall difference in likelihoods is driven by variant NC_000021.9:g.32602299G>C (rs202094637, 21:33974609G>C) linked to primary ciliary dyskinesia[50] where SB676 has allele reads of A = 0, C = 3, G = 6, T = 0 (LR=113.4); variant NC_000007.14:g.83961537G>A (rs138952094, 7:83590853G>A) reported as linked to delayed puberty[16] where SB605 has allele reads of A = 3, C = 0, G = 6, T = 0 (LR=67.8); variant NC_000005.10:g.112839514T>A (rs1801155, 5:112175211T>A) linked to cancer predisposition[10] where SB676 has allele reads of A = 4, C = 0, G = 0, T = 6 (LR=48.6); and variant NC_000022.11:g.50528591C>T (rs188 802138, 22:50967020C>T) linked to mitochondrial DNA depletion syndrome 1[16] where SB696 has allele reads of A = 0, C = 0, G = 0, T = 1 (LR = 2.7). We note that the interpretation of NC_000007.14:g.83961537G>A as pathogenic has been

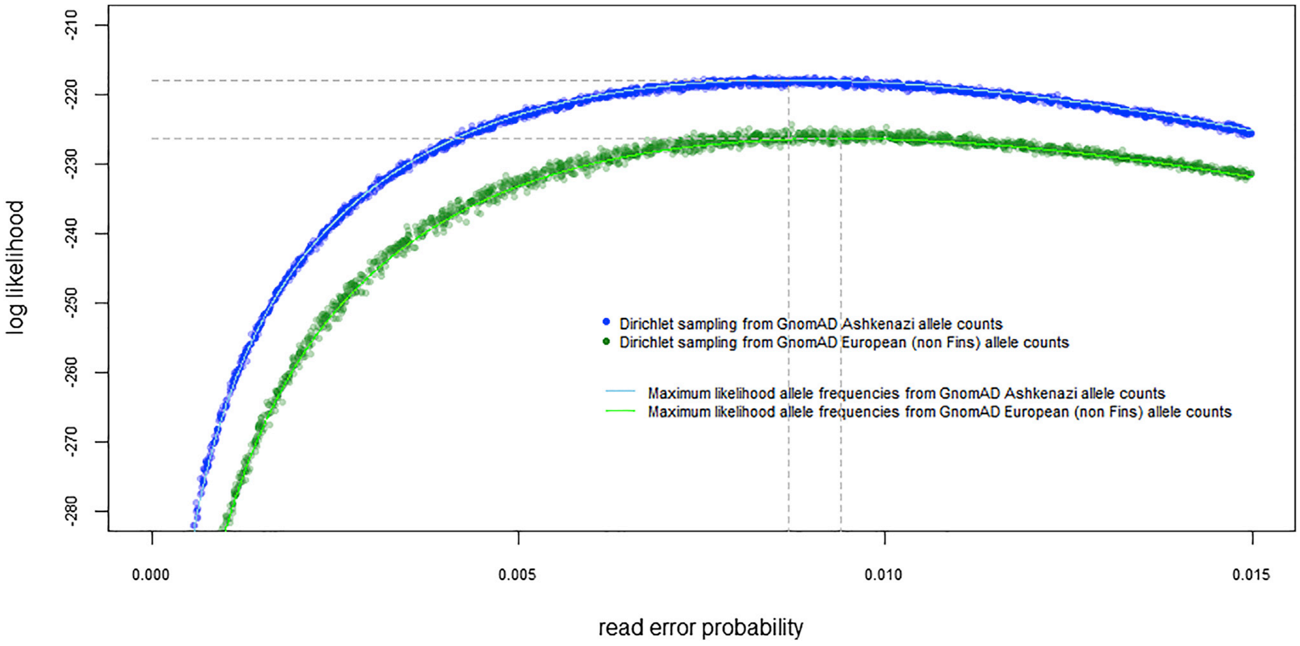

Figure 6. Determining the probability of observed genetic disorder reads in the Chapelfield sample, given different population origins

Log likelihoods compare the probability that the observed data were generated under a model of either the modern Ashkenazi population or the modern European (non-Finnish) population, using respective population allele frequencies in the gnomAD database. Each population model has its own parameter α, which determines the allele read error probability and is assumed constant across all loci. The maximum likelihood ratio indicates the data are 4,615 times more probable under the Ashkenazi model than the European model.

recently changed to uncertain on ClinVar,[53] but this does not affect the overall conclusion from our analyses that disease-associated alleles rose to frequencies similar to that in modern Ashkenazi populations by the 12th century.

DISCUSSION

We identify the presence of multiple related individuals at the Chapelfield site, with ancestry similar to modern Ashkenazi Jews, and a combined radiocarbon date of 1161–1216 calCE (95% confidence). These findings are consistent with accounts of the 1190 CE antisemitic attacks,[2] involving the targeting of households. It is therefore highly probable that the Chapelfield remains were those of victims of the 1190 CE riots, despite the challenges of associating archaeological sites with specific historical events. The red hair pigmentation prediction for SB604 is notable as medieval antisemitic tropes often incorporated an association between Jews and red hair.[47] Our results also indicate that Ashkenazim-associated disease alleles rose to near-modern frequencies prior to the 12th century CE. Since the majority of these alleles are at comparatively low frequencies in Sephardi Jews, the population bottleneck most likely to have resulted in elevated frequencies is one associated with the formation of Ashkenazim communities in northern Europe during the early medieval period.

STAR★METHODS

Detailed methods are provided in the online version of this paper and include the following:

- KEY RESOURCES TABLE
- RESOURCE AVAILABILITY
  - Lead contact
  - Materials availability
  - Data and code availability
- EXPERIMENTAL MODEL AND SUBJECT DETAILS
  - Description of archaeological and historical background to human remains
  - Background sampling information
- METHOD DETAILS
  - Radiocarbon dating
  - Ancient DNA extraction and sequencing
- QUANTIFICATION AND STATISTICAL ANALYSIS
  - DNA sequence data processing and alignment
  - Ancient DNA authentication and genotype calling
  - Sex and uniparental haplogroups
  - DNA fragmentation
  - Inferring population ancestry
  - Inferring pigmentation phenotypes
  - Inferring familial relationships and inbreeding
  - Analysis of Ashkenazi-associated mendelian disorders

SUPPLEMENTAL INFORMATION

Supplemental information can be found online at https://doi.org/10.1016/j.cub.2022.08.036.

ACKNOWLEDGMENTS

I.B., M.G.T., S.B., Y.D., and T.B. were supported by a Wellcome Trust Investigator Award (project no. 100713/Z/12/Z). Y.D. and M.G.T. received funding from the ERC Horizon 2020 research and innovation programme (grant

CellPress
OPEN ACCESS

agreement no. 788616 YMPACT), and A.T. and M.G.T. received funding from the ERC Horizon 2020 research and innovation programme (grant agreement no. 951385 COREX). R.M. is supported by an SSHRC doctoral studentship grant (G101449: Individual Life Histories in Long-Term Cultural Change). S.B. was supported by Illumina, who provided reagents for NovaSeq sequencing. The authors would like to thank Alan West and Tim Pestell at Norwich Castle Museum for their help with sampling and contextualizing the site, David Dobson at NPS Archaeology for Figure 1A, and Dr. Dean Irwin and Dr. Adam Rutherford for their comments on earlier versions of the manuscript. We wish to thank The Ashkenazi Genomes Consortium (TAGC) for providing us access to the most recent TAGC dataset and are grateful to Doron Behar and Shai Carmi for advice on access. The authors acknowledge the role of the Norwich Hebrew Congregation and the Office of the Chief Rabbi in supporting this project.

AUTHOR CONTRIBUTIONS

Conceptualization, I.B. and S.B.; methodology, I.B., S.B., T.B., Y.D., R.M., and M.G.T.; investigation, S.B. and T.B.; formal analysis, Y.D., T.B., M.G.T., A.T., W.S., and R.M.; writing – original draft, T.B., I.B., S.B., R.M., A.T., Y.D., and M.G.T.; writing – review & editing, T.B., I.B., S.B., R.M., A.T., Y.D., M.G.T., G.E., and S.C.; visualization, Y.D., T.B., A.T., W.S., and R.M.; supervision, I.B. and M.G.T.; funding acquisition, I.B., S.B., and M.G.T.

DECLARATION OF INTERESTS

The authors declare no competing interests.

INCLUSION AND DIVERSITY

One or more of the authors of this paper self-identifies as a member of the LGBTQ+ community.

Received: June 21, 2022
Revised: July 26, 2022
Accepted: August 12, 2022
Published: August 30, 2022

REFERENCES

1. Emery, G., Dobson, D., Hoggett, R., and Whitmore, D. (2010). A Medieval Mass Grave on the site of the Chapelfield Shopping Centre (NAU Archaeology).
2. Lipman, V.D. (1967). The Jews of Medieval Norwich (Jewish Historical Society of England).
3. Hillaby, J. (2013). Prelude and Postscript to the York Massacre: Attacks in East Anglia and Lincolnshire, 1190. In Christians and Jews in Angevin England: The York Massacre of 1190, Narratives and Contexts, S.R. Jones, and S. Watson, eds. (Melton: Boydell & Brewer), pp. 43–56.
4. Thomas of Monmouth. (2014). The Life and Passion of William of Norwich (Penguin Classics). Translated by M. Rubin.
5. Green, B., and Young, R.M.R. (1972). Norwich: the Growth of a City: A Brief History of the City (Norfolk Museums Service).
6. de Diceto, R. (1876). Historical works of Master Ralph de Diceto, Dean of London, W. Stubbs, ed. (Longmans).
7. Ostrer, H. (2001). A genetic profile of contemporary Jewish populations. Nat. Rev. Genet. *2*, 891–898.
8. Behar, D.M., Metspalu, E., Kivisild, T., Achilli, A., Hadid, Y., Tzur, S., Pereira, L., Amorim, A., Quintana-Murci, L., Majamaa, K., et al. (2006). The matrilineal ancestry of Ashkenazi Jewry: portrait of a recent founder event. Am. J. Hum. Genet. *78*, 487–497.
9. Atzmon, G., Hao, L., Pe'er, I., Velez, C., Pearlman, A., Palamara, P.F., Morrow, B., Friedman, E., Oddoux, C., Burns, E., and Ostrer, H. (2010). Abraham's children in the genome era: major Jewish diaspora populations comprise distinct genetic clusters with shared Middle Eastern Ancestry. Am. J. Hum. Genet. *86*, 850–859.
10. Carmi, S., Hui, K.Y., Kochav, E., Liu, X., Xue, J., Grady, F., Guha, S., Upadhyay, K., Ben-Avraham, D., Mukherjee, S., et al. (2014). Sequencing an Ashkenazi reference panel supports population-targeted personal genomics and illuminates Jewish and European origins. Nat. Commun. *5*, 4835.
11. Behar, D.M., Garrigan, D., Kaplan, M.E., Mobasher, Z., Rosengarten, D., Karafet, T.M., Quintana-Murci, L., Ostrer, H., Skorecki, K., and Hammer, M.F. (2004). Contrasting patterns of Y chromosome variation in Ashkenazi Jewish and host non-Jewish European populations. Hum. Genet. *114*, 354–365.
12. Myerowitz, R., and Costigan, F.C. (1988). The major defect in Ashkenazi Jews with Tay-Sachs disease is an insertion in the gene for the alpha-chain of beta-hexosaminidase. J. Biol. Chem. *263*, 18587–18589.
13. Beutler, E., Nguyen, N.J., Henneberger, M.W., Smolec, J.M., McPherson, R.A., West, C., and Gelbart, T. (1993). Gaucher disease: gene frequencies in the Ashkenazi Jewish population. Am. J. Hum. Genet. *52*, 85–88.
14. Roa, B.B., Boyd, A.A., Volcik, K., and Richards, C.S. (1996). Ashkenazi Jewish population frequencies for common mutations in BRCA1 and BRCA2. Nat. Genet. *14*, 185–187.
15. Struewing, J.P., Hartge, P., Wacholder, S., Baker, S.M., Berlin, M., McAdams, M., Timmerman, M.M., Brody, L.C., and Tucker, M.A. (1997). The risk of cancer associated with specific mutations of BRCA1 and BRCA2 among Ashkenazi Jews. N. Engl. J. Med. *336*, 1401–1408.
16. Rivas, M.A., Avila, B.E., Koskela, J., Huang, H., Stevens, C., Pirinen, M., Haritunians, T., Neale, B.M., Kurki, M., Ganna, A., et al. (2018). Insights into the genetic epidemiology of Crohn's and rare diseases in the Ashkenazi Jewish population. PLoS Genet. *14*, e1007329.
17. Klugman, S., and Gross, S.J. (2010). Ashkenazi Jewish screening in the twenty-first century. Obstet. Gynecol. Clin. North Am. *37*, 37–46.
18. Risch, N., de Leon, D., Ozelius, L., Kramer, P., Almasy, L., Singer, B., Fahn, S., Breakefield, X., and Bressman, S. (1995). Genetic analysis of idiopathic torsion dystonia in Ashkenazi Jews and their recent descent from a small founder population. Nat. Genet. *9*, 152–159.
19. Risch, N., Tang, H., Katzenstein, H., and Ekstein, J. (2003). Geographic distribution of disease mutations in the Ashkenazi Jewish population supports genetic drift over selection. Am. J. Hum. Genet. *72*, 812–822.
20. Kedar-Barnes, I., and Rozen, P. (2004). The Jewish people: their ethnic history, genetic disorders and specific cancer susceptibility. Fam. Cancer *3*, 193–199.
21. Slatkin, M. (2004). A population-genetic test of founder effects and implications for Ashkenazi Jewish diseases. Am. J. Hum. Genet. *75*, 282–293.
22. Behar, D.M., Yunusbayev, B., Metspalu, M., Metspalu, E., Rosset, S., Parik, J., Rootsi, S., Chaubey, G., Kutuev, I., Yudkovsky, G., et al. (2010). The genome-wide structure of the Jewish people. Nature *466*, 238–242.
23. Bray, S.M., Mulle, J.G., Dodd, A.F., Pulver, A.E., Wooding, S., and Warren, S.T. (2010). Signatures of founder effects, admixture, and selection in the Ashkenazi Jewish population. Proc. Natl. Acad. Sci. USA *107*, 16222–16227.
24. Mozersky, J. (2012). Risky genes: genetics, breast cancer and Jewish identity (Routledge).
25. Rotter, J.I., and Diamond, J.M. (1987). What maintains the frequencies of human genetic diseases? Nature *329*, 289–290.
26. Motulsky, A.G. (1995). Jewish diseases and origins. Nat. Genet. *9*, 99–101.
27. Weinryb, B.D. (1973). The Jews of Poland: a social and economic history of the Jewish community in Poland from 1100 to 1800 (Jewish Publication Society of America).
28. DellaPergola, S. (1997). Some fundamentals of Jewish demographic history. In Papers in Jewish Demography, S. DellaPergola, and J. Even, eds. (Jerusalem: Hebrew University).
29. Bronk Ramsey, C. (2009). Bayesian Analysis of Radiocarbon Dates. Radiocarbon *51*, 337–360.
30. Reimer, P.J., Austin, W.E.N., Bard, E., Bayliss, A., Blackwell, P.G., Bronk Ramsey, C., Butzin, M., Cheng, H., Edwards, R.L., Friedrich, M., et al.

(2020). The IntCal20 Northern Hemisphere Radiocarbon Age Calibration Curve (0–55 cal kBP). Radiocarbon *62*, 725–757.

31. Fu, Q., Mittnik, A., Johnson, P.L.F., Bos, K., Lari, M., Bollongino, R., Sun, C., Giemsch, L., Schmitz, R., Burger, J., et al. (2013). A revised timescale for human evolution based on ancient mitochondrial genomes. Curr. Biol. *23*, 553–559.
32. Link, V., Kousathanas, A., Veeramah, K., Sell, C., Scheu, A., and Wegmann, D. (2017). ATLAS: Analysis Tools for Low-depth and Ancient Samples. bioRxiv. https://doi.org/10.1101/105346.
33. Kistler, L., Ware, R., Smith, O., Collins, M., and Allaby, R.G. (2017). A new model for ancient DNA decay based on paleogenomic meta-analysis. Nucleic Acids Res. *45*, 6310–6320.
34. Heller, E.G. (2019). Western Ashkenazi Synagogues in Medieval and Early Modern Europe. In Jewish Religious Architecture: From Biblical Israel to Modern Judaism, S. Fine, ed. (Brill), pp. 169–183.
35. Mundill, R.R. (2010). The King's Jews: Money, Massacre and Exodus in Medieval England (Bloomsbury Publishing).
36. Haak, W., Lazaridis, I., Patterson, N., Rohland, N., Mallick, S., Llamas, B., Brandt, G., Nordenfelt, S., Harney, E., Stewardson, K., et al. (2015). Massive migration from the steppe was a source for Indo-European languages in Europe. Nature *522*, 207–211.
37. Xue, J., Lencz, T., Darvasi, A., Pe'er, I., and Carmi, S. (2017). The time and place of European admixture in Ashkenazi Jewish history. PLoS Genet. *13*, e1006644.
38. Kopelman, N.M., Stone, L., Hernandez, D.G., Gefel, D., Singleton, A.B., Heyer, E., Feldman, M.W., Hillel, J., and Rosenberg, N.A. (2020). High-resolution inference of genetic relationships among Jewish populations. Eur. J. Hum. Genet. *28*, 804–814.
39. Pereira, L., Richards, M., Goios, A., Alonso, A., Albarrán, C., Garcia, O., Behar, D.M., Gölge, M., Hatina, J., Al-Gazali, L., et al. (2006). Evaluating the forensic informativeness of mtDNA haplogroup H sub-typing on a Eurasian scale. Forensic Sci. Int. *159*, 43–50.
40. Brandstätter, A., Zimmermann, B., Wagner, J., Göbel, T., Röck, A.W., Salas, A., Carracedo, A., and Parson, W. (2008). Timing and deciphering mitochondrial DNA macro-haplogroup R0 variability in Central Europe and Middle East. BMC Evol. Biol. *8*, 191.
41. Behar, D.M., van Oven, M., Rosset, S., Metspalu, M., Loogväli, E.-L., Silva, N.M., et al. (2012). A "Copernican" reassessment of the human mitochondrial DNA tree from its root. Am. J. Hum. Genet. *90*, 675–684. https://doi.org/10.1016/j.ajhg.2012.03.002.
42. Agranat-Tamir, L., Waldman, S., Martin, M.A.S., Gokhman, D., Mishol, N., Eshel, T., Cheronet, O., Rohland, N., Mallick, S., Adamski, N., et al. (2020). The genomic history of the bronze age southern levant. Cell *181*, 1146–1157.e11.
43. Skourtanioti, E., Erdal, Y.S., Frangipane, M., Balossi Restelli, F., Yener, K.A., Pinnock, F., Matthiae, P., Özbal, R., Schoop, U.-D., Guliyev, F., et al. (2020). Genomic History of Neolithic to Bronze Age Anatolia, Northern Levant, and Southern Caucasus. Cell *181*, 1158–1175.e28.
44. Speidel, L., Cassidy, L., Davies, R.W., Hellenthal, G., Skoglund, P., and Myers, S.R. (2021). Inferring Population Histories for Ancient Genomes Using Genome-Wide Genealogies. Mol. Biol. Evol. *38*, 3497–3511.
45. Mallick, S., Li, H., Lipson, M., Mathieson, I., Gymrek, M., Racimo, F., Zhao, M., Chennagiri, N., Nordenfelt, S., Tandon, A., et al. (2016). The Simons Genome Diversity Project: 300 genomes from 142 diverse populations. Nature *538*, 201–206.
46. Chaitanya, L., Breslin, K., Zuñiga, S., Wirken, L., Pośpiech, E., Kukla-Bartoszek, M., Sijen, T., Knijff, P.d., Branicki, W., Liu, F., et al. (2018). The HIrisPlex-S system for eye, hair and skin colour prediction from DNA: Introduction and forensic developmental validation. Forensic Sci. Int. Genet. *35*, 123–135.
47. Livak, L. (2010). The Jewish Persona in the European Imagination (Stanford University Press).
48. Zlotogora, J. (2015). Mendelian Disorders Among Jews (Tel Aviv: Department of Community Genetics, Public Health Services, Ministry of Health, Israel). https://www.health.gov.il/Subjects/Genetics/Documents/book_jews.pdf.
49. Shi, L., Webb, B.D., Birch, A.H., Elkhoury, L., McCarthy, J., Cai, X., Oishi, K., Mehta, L., Diaz, G.A., Edelmann, L., and Kornreich, R. (2017). Comprehensive population screening in the Ashkenazi Jewish population for recurrent disease-causing variants. Clin. Genet. *91*, 599–604.
50. Wallace, S.E., and Bean, L.J.H. (2018). Resources for Genetics Professionals—Genetic Disorders Associated with Founder Variants Common in the Ashkenazi Jewish Population. In GeneReviews® [Internet] (University of Washington, Seattle).
51. Palamara, P.F., Lencz, T., Darvasi, A., and Pe'er, I. (2012). Length distributions of identity by descent reveal fine-scale demographic history. Am. J. Hum. Genet. *91*, 809–822.
52. Karczewski, K.J., Francioli, L.C., Tiao, G., Cummings, B.B., Alföldi, J., Wang, Q., Collins, R.L., Laricchia, K.M., Ganna, A., Birnbaum, D.P., et al. (2020). The mutational constraint spectrum quantified from variation in 141, 456 humans. Nature *581*, 434–443.
53. Landrum, M.J., Lee, J.M., Benson, M., Brown, G.R., Chao, C., Chitipiralla, S., Gu, B., Hart, J., Hoffman, D., Jang, W., et al. (2018). ClinVar: improving access to variant interpretations and supporting evidence. Nucleic Acids Res. *46*, D1062–D1067.
54. Reich, D. (2021). Allen Ancient DNA Resource version 44.3. https://reich.hms.harvard.edu/allen-ancient-dna-resource-aadr-downloadable-genotypes-present-day-and-ancient-dna-data.
55. Schubert, M., Lindgreen, S., and Orlando, L. (2016). AdapterRemoval v2: rapid adapter trimming, identification, and read merging. BMC Res. Notes *9*, 88.
56. Tarasov, A., Vilella, A.J., Cuppen, E., Nijman, I.J., and Prins, P. (2015). Sambamba: fast processing of NGS alignment formats. Bioinformatics *31*, 2032–2034.
57. Broad Institute (2020). Picard Tools version 2.23.3. http://broadinstitute.github.io/picard/.
58. McKenna, A., Hanna, M., Banks, E., Sivachenko, A., Cibulskis, K., Kernytsky, A., Garimella, K., Altshuler, D., Gabriel, S., Daly, M., and DePristo, M.A. (2010). The Genome Analysis Toolkit: a MapReduce framework for analyzing next-generation DNA sequencing data. Genome Res. *20*, 1297–1303.
59. Li, H. (2011). A statistical framework for SNP calling, mutation discovery, association mapping and population genetical parameter estimation from sequencing data. Bioinformatics *27*, 2987–2993.
60. Weissensteiner, H., Pacher, D., Kloss-Brandstätter, A., Forer, L., Specht, G., Bandelt, H.-J., Kronenberg, F., Salas, A., and Schönherr, S. (2016). HaploGrep 2: mitochondrial haplogroup classification in the era of high-throughput sequencing. Nucleic Acids Res. *44*. W58–63.
61. Ralf, A., González, D.M., Zhong, K., and Kayser, M. (2018). Yleaf: software for human Y-chromosomal haplogroup inference from next-generation sequencing data. Mol. Biol. Evol. *35*, 1820.
62. Wang, C., Zhan, X., Bragg-Gresham, J., Kang, H.M., Stambolian, D., Chew, E.Y., Branham, K.E., Heckenlively, J., FUSION Study, and Fulton, R., et al. (2014). Ancestry estimation and control of population stratification for sequence-based association studies. Nat. Genet. *46*, 409–415.
63. Patterson, N., Moorjani, P., Luo, Y., Mallick, S., Rohland, N., Zhan, Y., Genschoreck, T., Webster, T., and Reich, D. (2012). Ancient Admixture in Human History. Genetics *192*, 1065–1093.
64. Hanghøj, K., Moltke, I., Andersen, P.A., Manica, A., and Korneliussen, T.S. (2019). Fast and accurate relatedness estimation from high-throughput sequencing data in the presence of inbreeding. GigaScience *8*, giz034.
65. Chang, C.C., Chow, C.C., Tellier, L.C., Vattikuti, S., Purcell, S.M., and Lee, J.J. (2015). Second-generation PLINK: rising to the challenge of larger and richer datasets. GigaScience *4*, 7.
66. Ringbauer, H., Novembre, J., and Steinrücken, M. (2021). Parental relatedness through time revealed by runs of homozygosity in ancient DNA. Nat. Commun. *12*, 5425.

CellPress
OPEN ACCESS

67. Renaud, G., Hanghøj, K., Korneliussen, T.S., Willerslev, E., and Orlando, L. (2019). Joint estimates of heterozygosity and runs of homozygosity for modern and ancient samples. Genetics *212*, 587–614.

68. Dabney, J., Knapp, M., Glocke, I., Gansauge, M.-T., Weihmann, A., Nickel, B., Valdiosera, C., García, N., Pääbo, S., Arsuaga, J.-L., and Meyer, M. (2013). Complete mitochondrial genome sequence of a Middle Pleistocene cave bear reconstructed from ultrashort DNA fragments. Proc. Natl. Acad. Sci. USA *110*, 15758–15763.

69. Li, H., and Durbin, R. (2009). Fast and accurate short read alignment with Burrows-Wheeler transform. Bioinformatics *25*, 1754–1760.

70. Mathieson, I., Lazaridis, I., Rohland, N., Mallick, S., Patterson, N., Roodenberg, S.A., Harney, E., Stewardson, K., Fernandes, D., Novak, M., et al. (2015). Genome-wide patterns of selection in 230 ancient Eurasians. Nature *528*, 499–503.

71. Skoglund, P., Storå, J., Götherström, A., and Jakobsson, M. (2013). Accurate sex identification of ancient human remains using DNA shotgun sequencing. J. Archaeol. Sci. *40*, 4477–4482.

72. Mittnik, A., Wang, C.-C., Svoboda, J., and Krause, J. (2016). A molecular approach to the sexing of the triple burial at the upper paleolithic site of dolní věstonice. PLoS One *11*, e0163019.

73. Hofmanová, Z., Kreutzer, S., Hellenthal, G., Sell, C., Diekmann, Y., Díez-Del-Molino, D., van Dorp, L., López, S., Kousathanas, A., Link, V., et al. (2016). Early farmers from across Europe directly descended from Neolithic Aegeans. Proc. Natl. Acad. Sci. USA *113*, 6886–6891.

74. Lazaridis, I., Nadel, D., Rollefson, G., Merrett, D.C., Rohland, N., Mallick, S., Fernandes, D., Novak, M., Gamarra, B., Sirak, K., et al. (2016). Genomic insights into the origin of farming in the ancient Near East. Nature *536*, 419–424.

75. 1000 Genomes Project Consortium, Auton, A., Brooks, L.D., Durbin, R.M., Garrison, E.P., Kang, H.M., Korbel, J.O., Marchini, J.L., McCarthy, S., McVean, G.A., et al. (2015). A global reference for human genetic variation. Nature *526*, 68–74.

76. Waples, R.K., Albrechtsen, A., and Moltke, I. (2019). Allele frequency-free inference of close familial relationships from genotypes or low-depth sequencing data. Mol. Ecol. *28*, 35–48.

STAR★METHODS

KEY RESOURCES TABLE

REAGENT or RESOURCE	SOURCE	IDENTIFIER
**Biological samples**		
Human skeletal remains	This study	SB604
Human skeletal remains	This study	SB605
Human skeletal remains	This study	SB606
Human skeletal remains	This study	SB671
Human skeletal remains	This study	SB676
Human skeletal remains	This study	SB696
**Chemicals, peptides, and recombinant proteins**		
EDTA	Sigma Aldrich	Cat#03690-100ML
Tris-HCL	Fisher Scientific	Cat#10336763
Roche large volume viral kit, nucleic acid kit	Scientific Labs	Cat#5114403001
MinElute PCR Purification Kit	Qiagen	Cat#28006
UDG User Enzyme	New England Biolabs	Cat#M5505L
UGI	New England Biolabs	Cat#M0281S
10% Tween-20	Sigma Aldrich	Cat#P1379-25ML
AmpliTaq Gold	Fisher Scientific	Cat#N8080241
**Deposited data**		
The Genome Aggregation Database (gnomAD) v2.1.1	Karczewski et al.[52]	https://gnomad.broadinstitute.org/
The Ashkenazi Genomes Consortium (TAGC) dataset	Carmi et al.[10]	https://ega-archive.org/dacs/EGAC00001000151
Allen Ancient DNA Resource	Reich[54]	https://reich.hms.harvard.edu/allen-ancient-dna-resource-aadr-downloadable-genotypes-present-day-and-ancient-dna-data
**Software and algorithms**		
OxCal 4.4	Ramsey[29]	https://c14.arch.ox.ac.uk/oxcalhelp/hlp_contents.html
IntCal20 calibration curve	Reimer[30]	https://c14.arch.ox.ac.uk/oxcalhelp/hlp_curves.html
AdapterRemoval v2	Schubert et al.[55]	https://github.com/MikkelSchubert/adapterremoval
Sambamba	Tarasov et al.[56]	https://github.com/biod/sambamba
Picard Tools v2.23.3	Broad Institute[57]	https://broadinstitute.github.io/picard/
GATK v3.8	McKenna et al.[58]	https://github.com/broadinstitute/gatk
ATLAS	Link et al.[32]	https://bitbucket.org/wegmannlab/atlas/src/master/
ContamMix	Fu et al.[31]	https://github.com/DReichLab/adna-workflow
SAMtools v.1.9	Li[59]	https://github.com/samtools/samtools
Haplogrep	Weissensteiner et al.[60]	https://github.com/seppinho/haplogrep-cmd
Yleaf	Ralf et al.[61]	https://github.com/genid/Yleaf
LASER v.2.04	Wang et al.[62]	https://github.com/statgen/LASER
ADMIXTOOLS	Patterson et al.[63]	https://github.com/DReichLab/AdmixTools
Colate	Speidel et al.[44]	https://github.com/leospeidel/Colate
HIrisPlex-S	Chaitanya et al.[46]	https://hirisplex.erasmusmc.nl/
NgsRelate v.2	Hanghøj et al.[64]	https://github.com/ANGSD/NgsRelate
PLINK v1.09	Chang et al.[65]	https://www.cog-genomics.org/plink/
HapROH	Ringbauer et al.[66]	https://github.com/hringbauer/hapROH
ROHan	Renaud et al.[67]	https://github.com/grenaud/ROHan

RESOURCE AVAILABILITY

Lead contact

Further information and requests for resources and reagents should be directed to and will be fulfilled by the lead contact, Ian Barnes (i.barnes@nhm.ac.uk).

Materials availability

This study did not generate new unique reagents.

Data and code availability

- The accession number for the DNA sequences reported in this paper is ENA: PRJEB55223 (https://www.ebi.ac.uk/ena/data/view/PRJEB55223).
- Any additional information required to reanalyze the data reported in this paper is available from the lead contact upon request.

EXPERIMENTAL MODEL AND SUBJECT DETAILS

Description of archaeological and historical background to human remains

In 2004, workers constructing the Chapelfield shopping center (52.626N, 1.292E) in Norwich, Norfolk, UK, recovered human remains. Subsequent archaeological investigations by NAU Archaeology led to the discovery and excavation of a circular shaft, interpreted to have been originally used as a well. Further commingled human remains were found in partial articulation in a single mass burial deposit at the base of the well. The deposit was less than 0.5 m deep and 1 m in diameter, but contained the highly compacted remains of at least seventeen people. The well shaft had been truncated by the machine digger, which means that this number is probably an underestimate of the number of people originally deposited. Sediment surrounding the remains comprised soils from outside the well, suggesting bodies had been rapidly buried by soil after deposition. The well stratigraphy suggested that the well was disused and dry at the time the human remains were interred, and probably being used as a midden. Osteological analysis[1] identified at least six adults and eleven sub-adults. The subadult remains comprised at least one adolescent, two 10-15-year-olds, three 5-10-year-olds, three 3-5-year-olds and two children aged 0-3 years. The adult remains included both males and females. This overrepresentation of subadults suggests a catastrophic profile, where people of all ages had a similar risk of death, and the compaction of the remains suggested that they had been deposited in a single event. Patterns of skeletal articulation and completeness indicated that the skeletons were originally interred as complete, intact bodies, with little to no delay between their death and deposition. The Chapelfield burials thus appear to represent a catastrophic mortality event: a famine, epidemic or mass killing. In some cases the bones of the legs were higher up in the sediment than the skulls from the same skeletons, suggesting bodies had been deposited in the well head-first. There were no signs of trauma indicative of any mass killing event, beyond broken ribs that could have been produced when the bodies hit the bottom of the well, although it is possible they had been intentionally killed by a method which left no signs of trauma on the bones. There were no signs of skeletal trauma of a type commonly found in individuals who attempted to break a fall, providing some indication that the people had died before their bodies were deposited in the well. Some bones showed extremely localized brown-black or gray-blue discolorations and longitudinal splitting which can indicate exposure to high temperatures, although these changes can also be produced by diagenesis and mineral staining.

Background sampling information

In 2011, eight skeletal elements were sampled as part of the BBC series 'History Cold Case' (Series 2, Episode 3 '*The Bodies in The Well*'). DNA was extracted and the sections of the mitochondrial genome were targeted through PCR amplification. These methods and results are not described here, as they have been superseded by newer methods and data. In 2016 we accessed five additional skeletal elements, and these were sampled along with resampling of three of the most promising skeletal elements (based on the 2011 PCR results). DNA was extracted and NGS libraries constructed as described in the methods section below. In 2019 we were given permission to sample a further seventeen skeletal elements. DNA extraction and NGS library construction were also carried out as described below, with two additional libraries constructed for two of the most promising extracts from the 2016 sampling effort.

Ethical considerations

Rabbinical law prohibits exhumation of Jewish remans for most purposes. However, these remains were not thought to be those of Jews when excavated and initially analysed. Once the possibility that these individuals might have been Jewish was established, subsequent analyses were conducted with the cooperation and support of the Norwich Hebrew Congregation, and with the approval of the Office of the Chief Rabbi. The remains were reburied in 2013 at the Jewish cemetery in Norwich. There was a multi-faith reburial ceremony to accommodate the uncertainties surrounding the identity of the burials at that time. A commemorative plaque was also placed near the site of the well.

METHOD DETAILS

Radiocarbon dating

Two radiocarbon dates had previously been obtained at the University of Waikato (New Zealand) by Norvic Archaeology and reported in Emery et al.[1] Three previously unreported radiocarbon dates were commissioned by SHINE TV as part of the History Cold Case TV Series. The new dates were generated by Accelerator Mass Spectrometry (AMS) at the SUERC AMS facility, from extracted skeletal collagen, pretreated and reduced to graphite. All five radiocarbon dates were modelled in OxCal 4.4 using the IntCal20 calibration curve[29,30] The commingled skeletons were recovered from a single stratigraphic unit and were in partial articulation, suggesting that they had been buried in a single event soon after death.[1] The skeletons therefore meet assumptions required for their radiocarbon dates to be modelled with Bayesian inference as representing a single event using the Combine function in OxCal 4.4 to produce a refined posterior density estimate. The Combine model produces good agreement indices (Acomb = 75.8) and produces posterior density estimates for the deposition of the bodies of 1161-1216 calCE (95% confidence) or 1165-1207 calCE (68% confidence). Accelerator Mass Spectrometry (AMS) data generated is included in Table S1, and modelled likelihood distributions for these dating results are presented in Figure 1C.

Ancient DNA extraction and sequencing

Bone powder (27-67 mg) was removed using a Dremel drill at slow speed. DNA was extracted following a modified standard protocol after Dabney et al.[68] The protocol was modified by replacing Zymo-Spin V columns with Roche High Pure Viral Nucleic Acid Large Volume spin columns, and two final elution steps of 50 μl (total 100 μl) TET buffer (10 mM Tris·HCl, 1 mM EDTA, 0.05% Tween-20, pH 8.0). Dual indexed libraries were constructed based on standard double-stranded DNA protocols.[49,50] Prior to blunt end repair, the DNA extracts were partially UDG treated, 25 μl DNA extract, 3.6 μl USER enzyme (New England Biolabs), incubated at 37°C for 30 mins, followed immediately by adding 3.6 μl UGI enzyme (New England Biolabs) and incubation at 37°C for 30 mins. Reaction purification steps were carried out using minelute purification kits (Qiagen). Indexing PCR step used AmpliTaq Gold (Fisher Scientific) DNA polymerase. All pre PCR steps were carried out in the dedicated aDNA laboratory at the Natural History Museum, London (NHM). All 27 libraries (Data S1A) were screened on a NextSeq sequencing platform at the NHM using mid output 75 PE (150 cycles) kits. Nine libraries from six individuals were selected as the most likely to generate unique DNA reads (based on endogenous content and complexity), and deep sequenced on a NovaSeq 6000 S4 flow cell with v. 1 chemistry, for 200 cycles (Data S1B).

QUANTIFICATION AND STATISTICAL ANALYSIS

DNA sequence data processing and alignment

Sequencing data was analyzed with bioinformatics methods accounting for the properties of aDNA. Residual adapters were removed from both read pairs prior to merging using *AdapterRemoval*,[55] discarding reads shorter than 30 base pairs, trimming and collapsing forward and reverse reads with default parameters. Collapsed reads were aligned against the thousand genomes reference genome (hs37d5) with *bwa mem* and filtered for a minimal mapping quality of 30 with *samtools*.[59,69] BAM files were sorted with *sambamba,*[56] read groups set with *Picard*,[57] files merged and PCR duplicates marked with *sambamba*. *GATK v3.8*[70] was used for realignment around known InDels listed in the Broad '1000G phase1' and 'Mills and 1000G gold standard' resource files (provided as part of GATK). Additionally, we used a custom file with InDels causing diseases frequent in Jewish populations (Data S1D), from Carmi et al.[10]

Ancient DNA authentication and genotype calling

We inferred empirical post-mortem damage (PMD) patterns and recalibrated base quality scores with ATLAS.[33] PMD patterns at the first and last 50 base pairs of reads are shown in Figure S1 and confirm ancient DNA authenticity. Contamination estimates based upon mitochondrial genomes on a per fragment basis using *ContamMix*[31] (Figure S2) indicate no contamination. After computing PMD and recalibration patterns, genotypes were called with ATLAS, generating pseudo-haploid majority-allele calls (ATLAS options 'task=call method=majorityBase') for the sites covered by the 1240k capture array,[70] and diploid Bayesian maximum a posteriori calls (ATLAS options 'task=call method=Bayesian') for all sites with theta prior fixed at 0.001 (ATLAS options 'prior=theta fixedTheta=0.001') and equal base frequencies (ATLAS option 'equalBaseFreq'). In both cases two bases were trimmed from the ends of the reads. *SAMtools-mpileup*[59] was also used for studying observed disease alleles probabilistically.

Sex and uniparental haplogroups

Chromosomal sex was inferred from X and Y chromosomal read ratios following Skoglund et al.[71] (Figure S2). We note that individual SB696 did not yield a clear assignment using this method, as the R_y statistic minimally but confidently lies below the threshold for an XY call. We therefore computed the R_x statistic presented in Mittnik et al.,[72] which confidently classifies SB696 as male (R_x 95% CI [0.49, 0.55], Pearson's r 0.99).

Mitochondrial and Y-chromosomal haplogroups were inferred with *Haplogrep*[60] and *Yleaf*,[61] respectively (see Table S2). Table S3 provides further details of mitochondrial mutations observed among the chapelfield individuals (assigned through *Haplogrep*); this supports the association of observed uniparental haplotypes with Ashkenazi ancestry, discussed above.

DNA fragmentation

To further assess DNA degradation/fragmentation across our six similarly deposited individuals, we compared these individuals to a dataset of 95 archaeological individuals with varied depositional histories (age range ca. 40 to 10579 BP). We used the lambda parameter to estimate true fragment length[33] and control for differences in sequencing platforms (NextSeq and NovaSeq). We randomly selected six subsamples 1000 times from our pool of 95 archaeological individuals and plotted the standard deviation of lambda for each random subsample onto a density distribution. 95% confidence intervals were calculated and the standard deviation from our six samples added to the plot.

To assess whether the highly similar depositional histories of the Chapelfield individuals would lead to similar DNA degradation/fragmentation patterns across individuals we used the lambda parameter to estimate true fragment length.[33] When the standard deviation of six randomly sampled individuals (from a pool of 95 individuals with varied depositional histories (dated from approximately 40 to 10579 years BP) were plotted onto a density distribution, the standard deviation from our six Chapelfield individuals plotted outside the 95% confidence intervals of the distribution (Figure S1B). The result highlights that there is significantly more variation in lambda (fragment length) within the six Chapelfield individuals than there is between a set of randomly chosen samples over the last ~10,000 years. Deposition history does not therefore predict fragmentation of DNA on this timescale.

Inferring population ancestry

Explorative genetic ancestry analysis was performed by PCA using LASER v.2.04[73] following Hofmanová et al.,[73] projecting Chapelfield BAMs onto a reference space of modern Eurasian populations.[74] Populations used: Southern European (Italian North/South, Sicilian, Spanish/- North, Canary Islander, Maltese, Greek), Basque, Sardinian, Cypriot, Central European (Albanian, Bulgarian, Romanian, Hungarian, Croatian, Czech, German, French), Eastern European (Russian, Ukrainian, Belarussian, Polish, Sorb), Mordovian, Baltic and Finnish (Estonian, Lithuanian, Finnish), British Isles (English, Orcadian, Scottish, Irish/-Ulster, Shetlander), Scandinavian (Icelandic, Norwegian), Caucasian (Georgian, North Ossetian, Abkhasian, Chechen, Adygei, Lezgin, Kumyk, Balkar), West Asian (Turkish, Armenian), Iranian/-Bandari, Near Eastern (Palestinian, Druze, Jordanian), Ashkenazi-, Turkish-, and North African Jews (Libyan, Moroccan, Tunisian). This used pileup files (*SAMtools* v.1.9[56]) using filter criteria of minimum mapping quality 30 and minimum base quality 20. f_4 admixture proportions were computed with *qpAdm* from ADMIXTOOLS,[63] using default parameters and on pseudohaploid calls at the positions overlapping with the human origin/Illumina capture, on a non-related subset of Chapelfield individuals (SB604, SB605, SB676). Outgroups for *qpAdm* were individuals with labels Han, Karitiana, Mbuti, and Papuan[74] and ancient genomes Russia_MA1_HG.SG, Ethiopia_4500BP_published.SG, Belgium_UP_GoyetQ116_1_published, Russia_Ust_Ishim.DG, Spain_ElMiron, retrieved from the Allen Ancient DNA Resource.[54] Pairwise coalescence rates using published genomes (Data S1C) were estimated with *Colate.*[44] *Colate* input was precomputed from recalibrated BAM files (ATLAS option 'task=recal') of the Chapelfield individuals, using the provided SGDP[45] 'half_ne_fixed' mutation ages (*Colate* mode 'make_tmp'). Similarly, we precomputed *Colate* input from VCF files for the SGDP samples used, additionally specifying the 1000 Genomes accessible regions[75] as the target mask. Lastly, we estimated pairwise coalescence rates between each pair of samples (*Colate* mode 'mut'), with accessible regions mask for Chapelfield individuals, 20 bootstraps, and epoques determined by bins '2.92,4.52,0.4'.

Inferring pigmentation phenotypes

Pigmentation phenotypes were predicted based on the HIrisPlex-S method[46] on the three individuals with sufficient genomic coverage (SB604, SB605 and SB676, see Table S4). In case of missing genotypes in the VCF, we identify the corresponding positions in the recalibrated BAM files, and count one allele if the allele required by HIrisPlex-S is observed in at least one read (See Data S1H). We solely report the category with the highest probability if it is above 75%, and both the highest and second highest if the former is above 50% and the latter above 25%. See Table S2 in Chaitanya et al.[46] for the author's preliminary guide on how to interpret the skin pigmentation probability profiles. We obtained results only for the three individuals with sufficient genomic coverage.

Inferring familial relationships and inbreeding

To determine biological familial relationships among the six sequenced Chapelfield individuals we used the KING-robust method[76] implemented in *NgsRelate* v.2[64] to estimate R0, R1 and KING- robust kinship statistics. This approach is intended for sequencing data of such low coverage that accurate genotypes cannot necessarily be called.[76]

To study parental relatedness among the sequenced Chapelfield individuals we first studied runs of homozygosity using pseudohaploid data on the basis of a modern phased haplotype reference panel.[66] Inbreeding coefficients were additionally calculated on the basis of the fraction of the genome estimated to be within homozygous-by-descent segments using the sliding window approach implemented in PLINK v1.09.[65] Initially a MAF filter of 0.05 was applied, then data was pruned for linkage disequilibrium (command: PLINK –indep 50 2 2), before a sliding window of 50 SNPs was applied (command: PLINK –homozyg –homozyg-window-het 0 –homozyg-snp 50 –homozyg-kb 1 –homozyg- density 5000 –homozyg-gap 5000). Detected ROH lengths (in cM) greater than the threshold above were then used to calculate inbreeding coefficients (Table S5). However, only SB604 had sufficient coverage for the inbreeding coefficient to be confidently calculated using this approach. ROHs were further inferred using a panel of reference haplotypes using hapROH,[66] and ROHan[67] for the single individual with sufficient coverage (SB604).

Analysis of Ashkenazi-associated mendelian disorders

To investigate Ashkenazi-associated Mendelian Disorders among the Chapelfield individuals we first collated a dataset of 178 SNVs interpreted as associated with disorders observed in Ashkenazi populations (Data S1E, with sources detailed) based on published data.[10,16,50] InDel variants were detected by realignment (see above). We considered allele frequencies for these variants in gnomAD[52] and retained 159 loci where the population allele frequency for modern Ashkenazi Jewish (ASJ) was greater than for modern non-Finnish European (NFE) for disease-associated variants (Data S1F). We considered genotypes for these loci probabilistically, introducing a read error parameter α, defining the probability that a single allele is incorrectly read as one of the other three nucleotides. To determine the expected number of observed disease alleles at different rates of read error, datasets assuming ASJ and NFE population allele frequencies were simulated by sampling A,C,G,T nucleotides at each locus, for each individual from a multinomial distribution, using the observed total read depth as the number of trials. To calculate the exact probability of the observed allele reads, we applied a likelihood function utilizing α, summing the probability of the observed data for all 10^6 permutations of the ten possible genotypes at each locus.

Probabilistic inference of genotypes

In order to assess the frequency of disease alleles in the Chapelfield individuals, we needed to address two key problems associated with ancient DNA data. Firstly, read errors were likely to be present, such as observing nucleotide T at position 11:71146886 for individual SB604, which is not present in any modern population in the gnomAD database. Secondly, read-depths were low, varying from 16.1 reads per locus for SB604 to only 0.17 reads per locus for SB606, with zero reads at 39.6% of loci when considering all 6 individuals separately (see Figure S4). We addressed these problems by considering genotypes probabilistically (rather than making categorical calls) and introducing a read error parameter α, defining the probability that a single allele is incorrectly read as one of the other three nucleotides. This value is used globally (same value for each individual, and at each locus), and we assume symmetry between nucleotides, such that the probability of A incorrectly read as C is the same for all other nine pairwise errors. For example, we assume the probability of a true T being read as G is $\alpha/3$.

Simulating allele reads

A single simulated dataset was generated in a three stage process. Firstly, the gnomAD allele counts of A,C,G,T (from a proposed population at a specific locus) were used as shape parameters in the Dirichlet distribution (plus one additional count for each nucleotide, as a uniform prior), to generate a single set of four allele frequencies. Secondly, these proposed allele frequencies were modified by the proposed read error rate α, according to the formulas D1, where: freqs = a vector of the proposed frequencies of A,C,G,T at a locus (summing to 1); error = the proposed read error rate α. Thirdly, allele counts were randomly sampled from the multinomial distribution, where the total observed counts (across all four nucleotides) were used as the 'number of trials' parameter, and the allele frequencies (modified by α) were used as the multinomial probabilities.

Formulas D1 (in R code):

```
A <- freqs[1]*(1-error) + sum(freqs[c(2,3,4)])*error/3
C <- freqs[2]*(1-error) + sum(freqs[c(1,3,4)])*error/3
G <- freqs[3]*(1-error) + sum(freqs[c(1,2,4)])*error/3
T <- freqs[4]*(1-error) + sum(freqs[c(1,2,3)])*error/3
```

Likelihoods and likelihood ratios

Likelihoods were calculated using a four stage process that utilized the observed allele read counts, proposed population allele frequencies, and the read-error rate α. Firstly, for a single locus, we generated all 1,000,000 permutations of the six individuals' ten possible genotypes (AA, AC, AG, AT, CC, CG, CT, GG, GT, TT), and calculated the frequency of each genotype permutation, given the gnomAD population allele frequencies and assuming Hardy-Weinberg equilibrium. Where gnomAD data provided counts for exomes and genomes we used the combination (sum of counts) of both. Secondly, we calculated the likelihood of each individual's ten genotypes (again at a single locus), using a proposed read-error rate α and the observed allele counts in the multinomial distribution as specified in formulas M1. Thirdly, we summed all 1,000,000 permutations of these likelihoods, weighted by the frequency of each genotype permutation (since each permutation is a possible explanation of the observed data). Fourthly, we repeated for each of the 159 loci, with the α parameter fixed across all loci, and the overall product (under the assumption that loci are independent) provided the exact probability of the observed data, under a model of thegnomAD allele frequencies and a single α parameter. This approach deliberately avoids making any categorical genotype calls, and instead maintains probabilistic genotypes for downstream calculations. This is of particular value when analyzing aDNA where allele read depths are typically low and read errors high. In comparison, data with high read coverage and low read error rates can be assigned genotypes with such high confidence that the computational cost of this permutational method is not justified. Note, for computational efficiency, where two of the four possible nucleotides have a zero count, these can be aggregated into a single 'other' category requiring only 46,656 permutations of six genotypes (V1/V1, V1/V2, V1/V3, V2/V2, V2/V3, V3/V3), see formulas M2, and similarly where three nucleotides have zero counts, only 729 permutations of three genotypes need calculating (V1/V1, V1/V2 and V2/V2), see formulas M3. Where all four nucleotides have zero counts there is no information, and the likelihood equals 1.

Formulas (in R code):

```
p1 <- 1-error
p2 <- error/3
p3 <- 0.5 - p2
```

CellPress
OPEN ACCESS

Formulas M1 if 4 nucleotides have counts, all 10 genotypes need to be considered. Likewise if only 3 nucleotides have counts, the fourth remains a possibility if produced by a read error.

```
V1.V1 <- dmultinom(counts, prob=c(p1,p2,p2,p2))
V1.V2 <- dmultinom(counts, prob=c(p3,p3,p2,p2))
V1.V3 <- dmultinom(counts, prob=c(p3,p2,p3,p2))
V1.V4 <- dmultinom(counts, prob=c(p3,p2,p2,p3))
V2.V2 <- dmultinom(counts, prob=c(p2,p1,p2,p2))
V2.V3 <- dmultinom(counts, prob=c(p2,p3,p3,p2))
V2.V4 <- dmultinom(counts, prob=c(p2,p3,p2,p3))
V3.V3 <- dmultinom(counts, prob=c(p2,p2,p1,p2))
V3.V4 <- dmultinom(counts, prob=c(p2,p2,p3,p3))
V4.V4 <- dmultinom(counts, prob=c(p2,p2,p2,p1))
```

Formulas M2 if only 2 nucleotides have counts, the remaining two can be combined into a single ‘other’, so that 6 genotypes need to be considered:

```
V1.V1 <- dmultinom(counts, prob=c(p1,p2,p2+p2))
V1.V2 <- dmultinom(counts, prob=c(p3,p3,p2+p2))
V1.V3 <- dmultinom(counts, prob=c(p3,p2,p3+p2))
V2.V2 <- dmultinom(counts, prob=c(p2,p1,p2+p2))
V2.V3 <- dmultinom(counts, prob=c(p2,p3,p3+p2))
V3.V3 <- dmultinom(counts, prob=c(p2,p2,p1+p2))
```

Formulas M3 if only 1 nucleotide has counts, the remaining 3 can be combined into a single ‘other’, so that only 3 genotypes need to be considered:

```
V1.V1 <- dmultinom(counts, prob=c(p1,p2+p2+p2))
V1.V2 <- dmultinom(counts, prob=c(p3,p3+p2+p2))
V2.V2 <- dmultinom(counts, prob=c(p2,p1+p2+p2))
```

Our method calculates likelihoods under the assumption that the six individuals are randomly sampled from a proposed population, and therefore does not take into account relatedness. In the case of these particular data, this assumption has a conservative influence on the likelihood ratio for the following reason. The overwhelming majority of the likelihood ratio is driven by variants that are private to a single individual (SB676 21-33974609-G-C LR=113.4; SB605 7-83590853-G-A LR=67.8; SB676 5-112175211-T-A LR=48.6; SB696 22-50967020-C-T LR=2.7), which removes any influence of relatedness on the likelihood ratios. Four further non-private disease alleles were observed in SB604 and SB676 14-94770808-C-T LR=0.954; 14-97342370-C-T LR=1.079; 21-43808633-C-A LR=0.863; 21-45713715-C-T LR=0.870), but since the likelihood ratios at these loci overall slightly favor the european population (less than 1), adjusting for relatedness would have the effect of slightly increasing the likelihood ratio. In any case, our familial relationship analysis did not find a close relationship between SB604 and SB676 that would justify such an adjustment. In contrast, the closest relationships identified were between siblings SB605, SB606 and SB671 who had no disease alleles in common.

Current Biology, Volume *32*

Supplemental Information

Genomes from a medieval mass burial show Ashkenazi-associated hereditary diseases pre-date the 12th century

Selina Brace, Yoan Diekmann, Thomas Booth, Ruairidh Macleod, Adrian Timpson, Will Stephen, Giles Emery, Sophie Cabot, Mark G. Thomas, and Ian Barnes

A

B

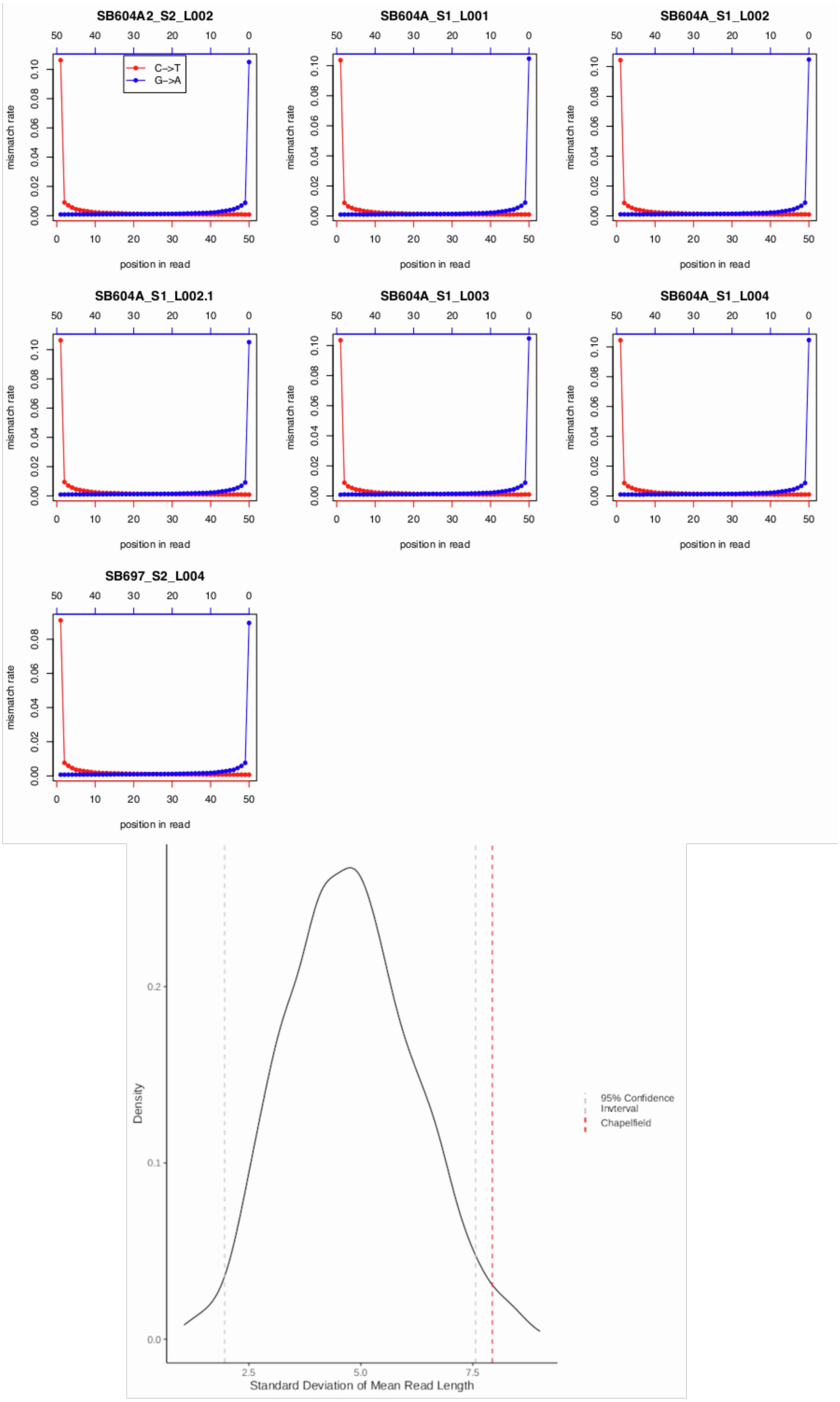

SB604A2_S2_L002
SB604A_S1_L001
SB604A_S1_L002
SB604A_S1_L002.1
SB604A_S1_L003
SB604A_S1_L004
SB697_S2_L004
C->T
G->A
mismatch rate
position in read
95% Confidence Invterval
Chapelfield
Density
Standard Deviation of Mean Read Length

Figure S1. Analysis of post-mortem damage patterns to confirm authenticity of aDNA, related to STAR Methods. (A) Misincorporation rates at DNA strand ends for seven sequencing libraries for the individual SB604, showing characteristic 'ancient' damage profiles in all libraries. These show cytosine to thymine and guanine to adenine misincorporation patterns at the first and last 50 base pairs of reads respectively for a subset of the sequenced libraries, confirming aDNA authenticity. Misincorporation rates were generated in ATLAS[S1]. (B) Standard deviation of mean read length for six randomly sampled ancient genomes compared with the Chapelfield individuals. This was investigated using the lambda parameter to estimate true fragment length[S2]; this indicates that depositional history is not a predictor of fragment length.

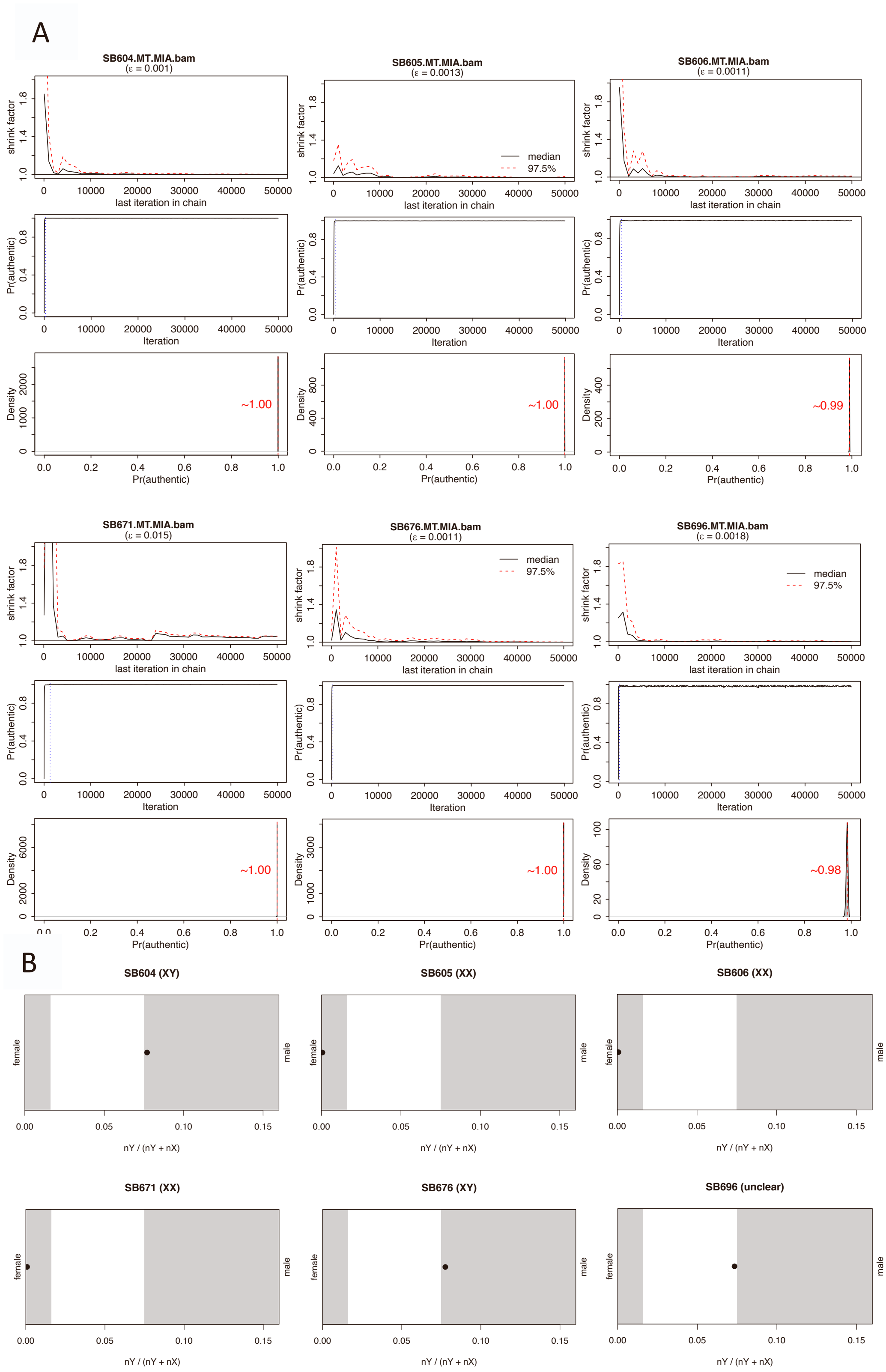
A
SB604.MT.MIA.bam
(ε = 0.001)
SB605.MT.MIA.bam
(ε = 0.0013)
SB606.MT.MIA.bam
(ε = 0.0011)
shrink factor
last iteration in chain
median
97.5%
Pr(authentic)
Iteration
Density
~1.00
~1.00
~0.99
SB671.MT.MIA.bam
(ε = 0.015)
SB676.MT.MIA.bam
(ε = 0.0011)
SB696.MT.MIA.bam
(ε = 0.0018)
~1.00
~1.00
~0.98
B
SB604 (XY)
SB605 (XX)
SB606 (XX)
SB671 (XX)
SB676 (XY)
SB696 (unclear)
female
male
nY / (nY + nX)

Figure S2. Basic bioinformatics analyses for mitochondrial sequence contamination and chromosomal sex, related to STAR Methods. (A) Results from mitochondrial contamination estimates for each sequenced individual. These indicate no mitochondrial sequence contamination, and were estimated through ContamMix[S3]. (B) Plots showing chromosomal sex of each individual based on ratios of X and Y chromosome sequences. These were predicted following the approach of Skoglund et al.[S4]. Shaded areas indicate female and male assignation; we also computed the R_x statistic from Mittnik et al.[S5], which confidently identified SB696 as male.

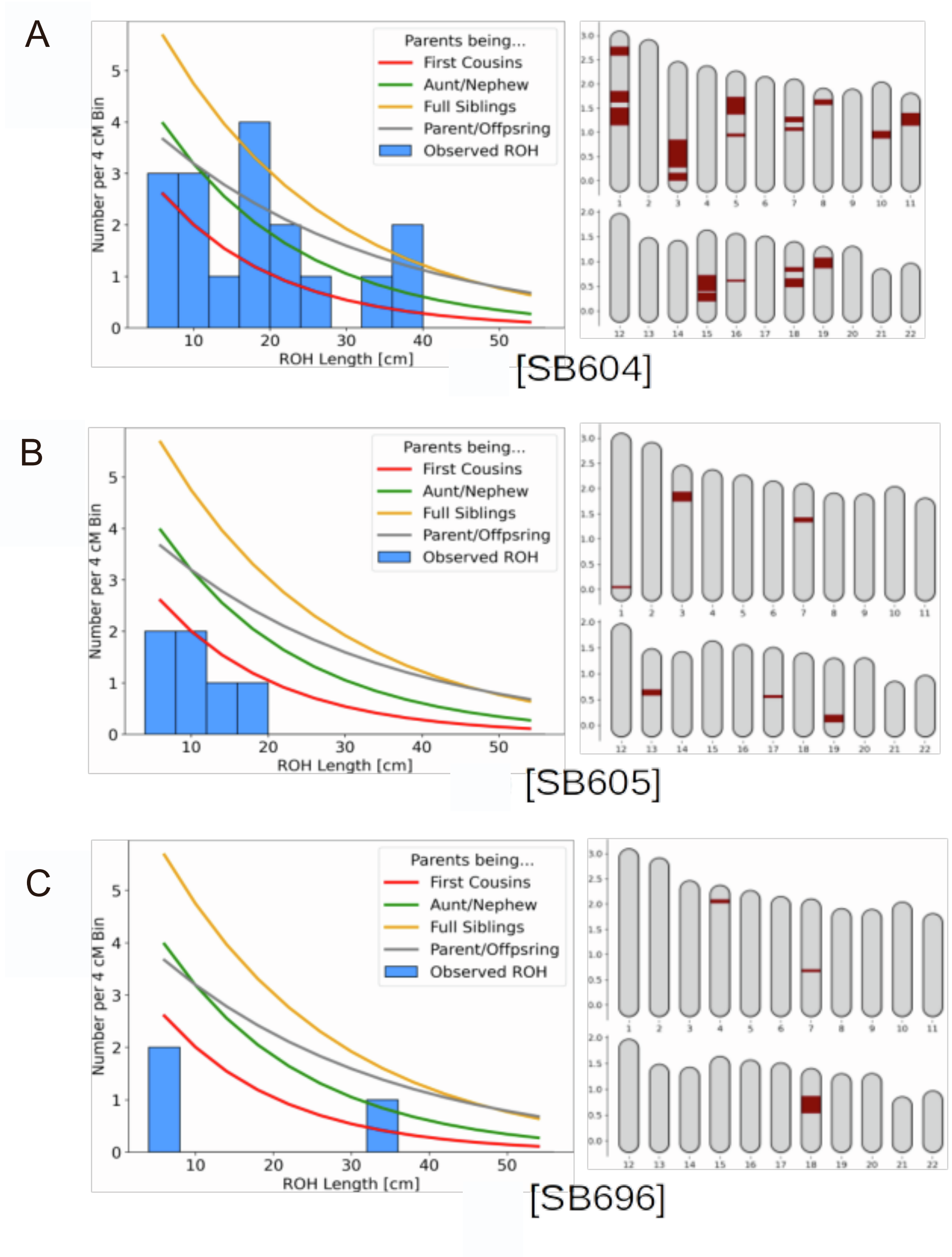

Figure S3. Runs of homozygosity inferred using hapROH, related to Figure 2. (A) Distribution of ROH lengths for SB604 compared with certain inbreeding scenarios (left), generated in hapROH[S6], and locations of large inferred ROHs on autosomes. The reference inbreeding scenarios overlain are generated automatically in hapROH based on calculations detailed in Ringbauer et al.[S6]. (B) As above for SB605. (C) As above for SB696.

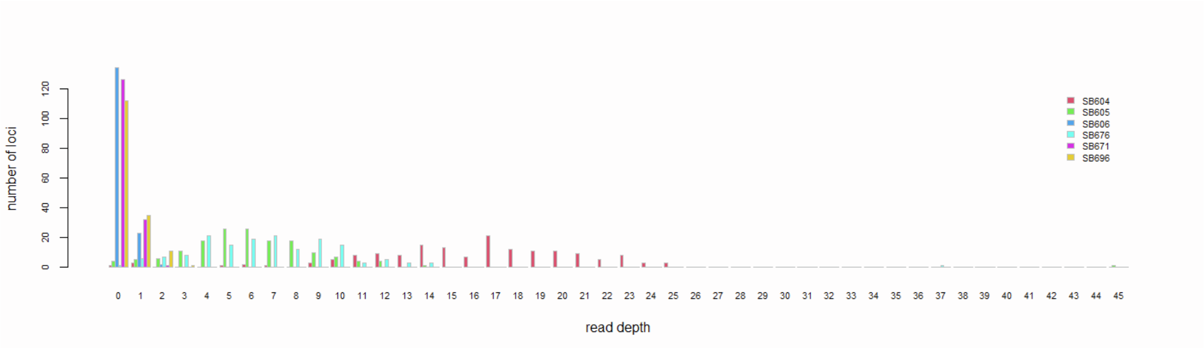

Figure S4. Read depth per disorder-associated loci for each sample, related to Figure 4 and STAR Methods. SB604 yielded the best read depth, averaging 16 reads per locus and zero reads at only 1 locus. SB606 yielded the poorest read depth, averaging 0.17 reads per locus with zero reads at 134 loci.

Sk Number (Deposit)	Lab Number	δ13C (AMS)	δ15N (AMS)	%C	%N	C:N Ratio	Radiocarbon Determination	Error
Sk 78	Wk16920	-18.3	13.14	45.5	16.2	3.28	875	34
Sk 62	Wk16919	-18.4	12.05	46.1	16.6	3.2	928	32
	SUERC-33391	-	-	-	-	-	910	30
	SUERC-33282	-	-	-	-	-	845	30
	SUERC-33281	-	-	-	-	-	850	30

Table S1. Radiocarbon date data, related to Figure 1C. This comprises Accelerator Mass Spectrometry (AMS) results for the total of five radiocarbon samples. Of these, two were reported by Emery et al.[S7] (Wk16920 and Wk16919) and the remaining three were commissioned by SHINE TV as part of the History Cold Case TV Series at the SUERC radiocarbon dating laboratory.

	Mitochondrial haplogroup	Y chromosome haplogroup
**SB604**	J1c5c1	J1a2a1a2d2b2a2b
**SB605**	H5c2	*NA*
**SB606**	H5c2	*NA*
**SB671**	H5c2	*NA*
**SB676**	H3w	E1b1b1b2a1b1a
**SB696**	U6a1b1b	T1a1a

Table S2. Uniparental haplogroups from HaploGrep2 and Yleaf, related to STAR Methods. Mitochondrial haplogroups were inferred using HaploGrep 2[S8] and Y chromosome haplogroups using Yleaf[S9].

	Mitochondrial haplogroup	Range	Quality	Global private mutations	Local private mutations	Assumed back mutations or missing
**SB604**	J1c5c1	1-16569	94.84%	3107T, 4490A, 4510G, 4687A, 5180, 8898A		4216, 7028, 8860
**SB605**	H5c2	1-16569	77.44%	3107T, 5177T, 7494A, 9081	3360, 5892	4769, 6776, 8860
**SB606**	H5c2	1-16569	78.69%	3107T, 4130A, 4134T, 4139A, 4141A, 4153T, 4165A, 7501G, 7541, 7634, 8919	4721, 8911	4769, 8860, 16304
**SB671**	H5c2	1-16569	72.34%	3107c, 4700, 5170, 5185, 5238, 5720, 5736, 5895, 5897, 6042A, 6107, 6877, 7096, 7504. 7550, 7552, 7566T, 7576, 7592, 7596A, 7627, 7732, 7756, 8897, 8903	1185, 4129, 4707, 4709, 7082, 7607	8860, 16304
**SB676**	H3w	1-16569	73.26%	3107T, 5177T, 7494A, 9081	3360, 5892	4769, 5892
**SB696**	U6a1b1b	1-16569	90.21%	4729A, 8001T	7696	4769, 7028, 8860, 11176

Table S3. Details of observed mitochondrial haplogroups and mutations, related to STAR Methods. These constitute results of classification using HaploGrep 2[S8] .

	PBlueEye	PIntermediateEye	PBrownEye	PRedHair	PBlondHair	PBrownHair	PBlackHair	PLightHair	PDarkHair	PVeryPaleSkin	PPaleSkin	PIntermediateSkin	PDarkSkin	PDarktoBlackSkin
**SB 604**	0.9110910019	0.05659703369	0.03231196441	0.9999541868	4.33E-05	1.33E-06	1.16E-06	0.9436118803	0.05638811966	0.02943904649	0.4400186053	0.5277118875	0.002826723683	3.74E-06
**SB 605**	0.000271927178	0.01339029227	0.9863377806	0.001123064907	0.06507180104	0.7585584683	0.1752466657	0.2407708189	0.7592291811	0.01111802015	0.05286550598	0.6327007046	0.2988146386	0.004501130685
**SB 676**	0.05032274533	0.1135511369	0.8361261178	0.004027658594	0.357854928	0.5652752736	0.07284213988	0.802023479	0.197976521	0.009700711401	0.3535418605	0.63433943	0.002399310288	1.87E-05

Table S4. Probabilities of phenotypes inferred with HirisPlexS, related to STAR Methods. Results are only reported for the three individuals with sufficient genomic analysis for this approach.

**Sample**	***f***	**Avg. autosomal depth**
SB604	0.2064325334	13.81x
SB605	0.09459731039	4.78x
SB606	0	0.16x
SB671	0	0.18x
SB676	0.1245771792	6.03x
SB696	0	0.31x

Table S5. Inbreeding coefficients (*f*) calculated using estimates of homozygous-by-descent segments in PLINK, related to Figure 2 and STAR Methods. For this analysis, only SB604 has sufficient genomic coverage for confident inferences to be drawn.

Supplemental References

S1. Link, V., Kousathanas, A., Veeramah, K., Sell, C., Scheu, A., and Wegmann, D. (2017). ATLAS: Analysis Tools for Low-depth and Ancient Samples. bioRxiv, 105346.

S2. Kistler, L., Ware, R., Smith, O., Collins, M., and Allaby, R.G. (2017). A new model for ancient DNA decay based on paleogenomic meta-analysis. Nucleic Acids Res. *45*, 6310–6320.

S3. Fu, Q., Mittnik, A., Johnson, P.L.F., Bos, K., Lari, M., Bollongino, R., Sun, C., Giemsch, L., Schmitz, R., Burger, J., et al. (2013). A revised timescale for human evolution based on ancient mitochondrial genomes. Curr. Biol. *23*, 553–559.

S4. Skoglund, P., Storå, J., Götherström, A., and Jakobsson, M. (2013). Accurate sex identification of ancient human remains using DNA shotgun sequencing. J. Archaeol. Sci. *40*, 4477–4482.

S5. Mittnik, A., Wang, C.-C., Svoboda, J., and Krause, J. (2016). A Molecular Approach to the Sexing of the Triple Burial at the Upper Paleolithic Site of Dolní Věstonice. PLoS One *11*, e0163019.

S6. Ringbauer, H., Novembre, J., and Steinrücken, M. (2021). Parental relatedness through time revealed by runs of homozygosity in ancient DNA. Nat. Commun. *12*, 5425.

S7. Emery, G., Dobson, D., Hoggett, R., and Whitmore, D. (2010). A Medieval Mass Grave on the site of the Chapelfield Shopping Centre, Norwich (NAU Archaeology).

S8. Weissensteiner, H., Pacher, D., Kloss-Brandstätter, A., Forer, L., Specht, G., Bandelt, H.-J., Kronenberg, F., Salas, A., and Schönherr, S. (2016). HaploGrep 2: mitochondrial haplogroup classification in the era of high-throughput sequencing. Nucleic Acids Res. *44*, W58–63.

S9. Ralf, A., Montiel González, D., and Zhong, K. (2018). Yleaf: software for human Y-chromosomal haplogroup inference from next-generation sequencing data. Mol. Biol.
